# Decoupling the Roles of Ligand-Field and Charge-Transfer Excitations on the Photocarrier Yield in Transition-Metal Oxides: Insights from MTiO_3_ (M = Ni, Co, Mn)

**DOI:** 10.1021/acs.inorgchem.6c01924

**Published:** 2026-10-08

**Authors:** Ron Fishov, Yotam Engel, Rohit Kumar Saini, Sa’ar Shor Peled, Kumaraswamy Miriyala, Daniel A. Grave

**Affiliations:** † Department of Materials Engineering, 26732 Ben-Gurion University of the Negev, Beer Sheva 8410500, Israel; ‡ Ilse Katz Institute for Nanoscale Science and Technology, 26732 Ben-Gurion University of the Negev, Beer Sheva 8410500, Israel

## Abstract

Open d-shell transition-metal oxides are widely studied as photoactive materials for solar-energy conversion but typically exhibit low yields of mobile photocarriers. This inefficiency has been linked to ligand-field (LF) states that induce ultrafast relaxation, although their impact across open d-shell oxides remains poorly understood. Here, epitaxial ilmenite-type MTiO_3_ (M = Ni, Co, Mn) thin films were stabilized by pulsed laser deposition. In this isostructural series, the M^2+^ cation tunes the d-electron configuration and the energetic separation and spectral overlap of LF and charge-transfer excitations. Combined optical and time-resolved microwave conductivity measurements, benchmarked against rutile TiO_2_ as a d^0^ reference, together with photoelectrochemical measurements of the ilmenites, reveal reduced yield-mobility products compared to TiO_2_. NiTiO_3_ and CoTiO_3_ show measurable photoconductivity only under UV charge-transfer excitation, whereas MnTiO_3_, dominated by a dense LF manifold, exhibits a negligible response. These results indicate that mobile charge-carrier generation contributing to photoelectrochemical activity originates from UV charge-transfer excitations, likely dominated by O^2–^ → Ti^4+^ ligand-to-metal charge-transfer (LMCT) excitation into Ti 3d-derived states, while LF states primarily act as relaxation pathways that do not yield mobile carriers on nanosecond time scales. These findings decouple the roles of distinct optical excitations in governing photocarrier localization in open d-shell oxides.

## Graphical abstract

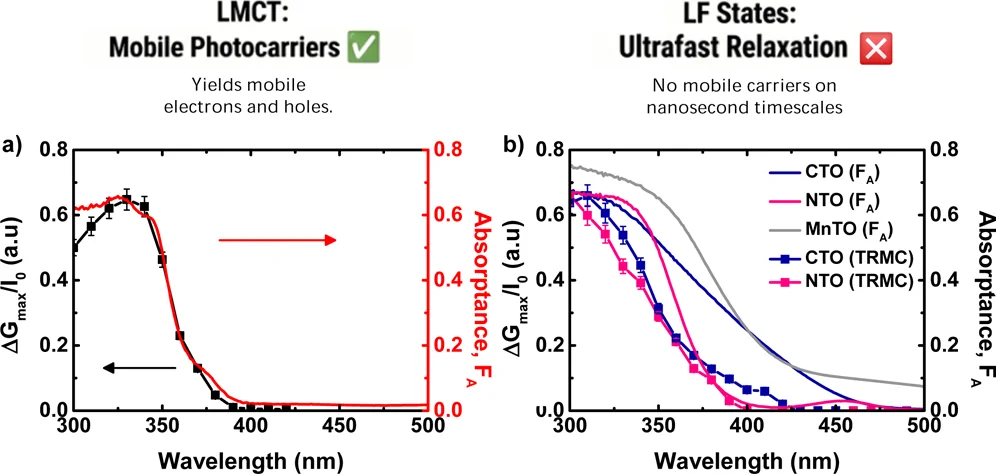

## 1 Introduction

Transition-metal oxides (TMOs) are promising candidates for solar-driven photoelectrochemical (PEC) water splitting.^1^ Champion photoelectrodes of transition metal-oxides with empty (d^0^) and/or full (d^10^) d-shell configurations, as seen in materials such as BiVO_4_ and TiO_2_, exhibit impressively high internal quantum efficiencies (IQEs) exceeding 90%.^2,3^ Regrettably, their wide band gaps preclude their viability for solar harvesting applications. In contrast, TMOs suitable for photoelectrodes generally possess open d shell configurations (d^*n*^, 0 < *n* < 10). Among the best performing open d-shell materials is hematite, α-Fe_2_O_3_, but even state-of-the-art photoanodes based on this material have reached IQEs of only 50%, significantly lower than their closed or empty d-shell counterparts such as TiO_2_.^4^

Recently, it has been suggested that a subunity yield of photogenerated mobile charge carriers is responsible for the poor photoconversion efficiency of hematite and other open d-shell materials.^5,6^ This is manifested in the wavelength-dependent behavior of the external quantum efficiency (EQE) of hematite, which has long been observed to decrease with wavelength at a rate that is faster than would be predicted by the decreasing optical absorption.^7,8^ To explain this behavior, Hayes et al. suggested that only ligand-to-metal charge-transfer (LMCT) transitions are believed to generate carriers that contribute to photocurrent while the highly localized ligand-field (LF) transitions do not.^9^ In a more recent study, Sachs et al. demonstrated through ultrafast transient absorption spectroscopy that LF states induce ultrafast relaxation pathways, resulting in lower carrier yields in open d-shell materials as compared to d^0^ or d^10^ materials.^10^ Among the open d-shell materials studied, hematite was found to have the highest carrier yields, attributed to spin-forbidden relaxation from charge-transfer states into the LF manifold, which partially mitigates this loss pathway. These findings highlight the critical role that different types of electronic excitations play in governing charge-carrier dynamics in TMOs. Indeed, it has recently been suggested that the transport lengths of holes are also dictated by the nature of the optical transitions in various TMOs.^11^ Despite these advances, it remains unclear what governs carrier yields and their spectral dependence among open d-shell TMOs with different electronic configurations.

In this work, the ilmenite MTiO_3_ (M = Ni, Co, Mn) system is used to systematically investigate how electronic configuration influences the optoelectronic and PEC properties of open d-shell oxides. The ilmenite system provides a model platform for such an investigation for several reasons. First, the divalent M^2+^ cation can be easily substituted, enabling systematic comparison across different transition metal oxides with the same crystal structure. Second, although earlier literature suggested that ilmenites can exhibit a broad nonstoichiometry range, allowing variations in the Ni/Ti ratio,^12^ more recent findings indicate that apparent deviations often arise from the formation of epitaxial vertically aligned TiO_2_ nanocolumns that alter the overall film stoichiometry.^13^ This highlights the importance of precise phase control when probing intrinsic optoelectronic properties. Third, in contrast to hematite where LF and LMCT transitions possess significant spectral overlap,^9^ ilmenites, due to their larger band gaps, exhibit a greater energetic separation between LF and charge-transfer transitions,^14–18^ facilitating clearer insight into the role of distinct electronic excitations in charge-carrier dynamics. Finally, MTiO_3_ (M = Ni, Co, Mn) compounds can be grown epitaxially on Nb:SnO_2_ ^19^ and sapphire,^12,20,21^ enabling high-quality crystalline films that allow direct correlation between structural characteristics, optoelectronic properties, and PEC performance.

To assess how distinct optical excitations contribute to mobile charge-carrier generation, we synthesized epitaxial NiTiO_3_ (NTO), CoTiO_3_ (CTO), and MnTiO_3_ (MnTO) thin films by pulsed laser deposition (PLD) and examined their structural, optical, PEC, and time-resolved microwave conductivity (TRMC) responses. By combining excitation-energy-dependent TRMC measurements with optical spectroscopy and EQE analysis, we identify the excitation pathways responsible for mobile carrier generation in this isostructural materials family and clarify the role of LF states in limiting carrier yield in open d-shell oxides.

## 2 Results and Discussion

### 2.1 Structural Characterization of Epitaxial MTiO_3_ Thin Films

To enable a comprehensive optoelectronic evaluation, the ilmenite thin films were grown epitaxially on two substrates. For TRMC measurements, the films were grown on single-crystal *c*-plane sapphire Al_2_O_3_(0001) substrates. For PEC measurements, the films were grown on Nb-doped SnO_2_ (Nb:SnO_2_)-coated sapphire. Nb:SnO_2_ is a transparent current collector with high electrical conductivity and transparency suitable for use as a bottom electrode for PEC measurements. By carefully refining the deposition parameters, epitaxial phase-pure ilmenite films were stabilized on both substrates as described below.

NTO thin films were deposited on *c*-plane sapphire substrates under 10 mTorr oxygen pressure and 800 °C. Initial high-resolution X-ray diffraction (HR-XRD) measurements did not show (003) and (009) peaks, which are characteristic of the ordered ilmenite phase, suggesting that the films instead crystallized in a metastable corundum-type structure (*R*3̅*c* space group). In this configuration, Ni^2+^ and Ti^4+^ cations are randomly distributed within the octahedral sites rather than arranged in the alternating layers that define the ilmenite structure.^20^ To achieve proper ordering, the films were postannealed in an open-air oven at 750 °C for 1 h. Postannealing HR-XRD θ–2θ out-of-plane diffraction pattern revealed the (003) and (009) peaks, confirming a well-ordered ilmenite structure (Figure 1a). Parts a and b of Figure S1 show the off-axis φ scans of the (1 0 10) reflections for the preannealing and postannealing, respectively, confirming the epitaxial in-plane alignment. The preannealing scan displays 6-fold rotational symmetry, where three reflections align with the substrate peaks, while the other three are rotated by 60°. This unexpected 60° rotation may result from the existence of twinning defects due to the high mismatch between the film and the substrate.^22^ After annealing, the film no longer shows 6-fold symmetry, but rather the expected 3-fold symmetry, indicating that the twinning defect has been eliminated. The rocking curves for the NTO preannealing and postannealing samples are shown in Figure S1c,d, respectively. Both curves display a narrow specular component and a weak broad diffuse component. The narrow component retains the same full width at half-maximum (fwhm) of 0.05°, whereas the weak broad diffuse component decreases from 0.39° before annealing to 0.27° after annealing. The narrow peak indicates regions of the film that are well-ordered over long distances, while the broad diffuse scattering suggests areas with slight rotational disorder, likely near the interface.^23^ After annealing, the narrow component becomes stronger relative to the broad component, indicating an improvement in the film’s long-range structural order.

**Figure 1:**
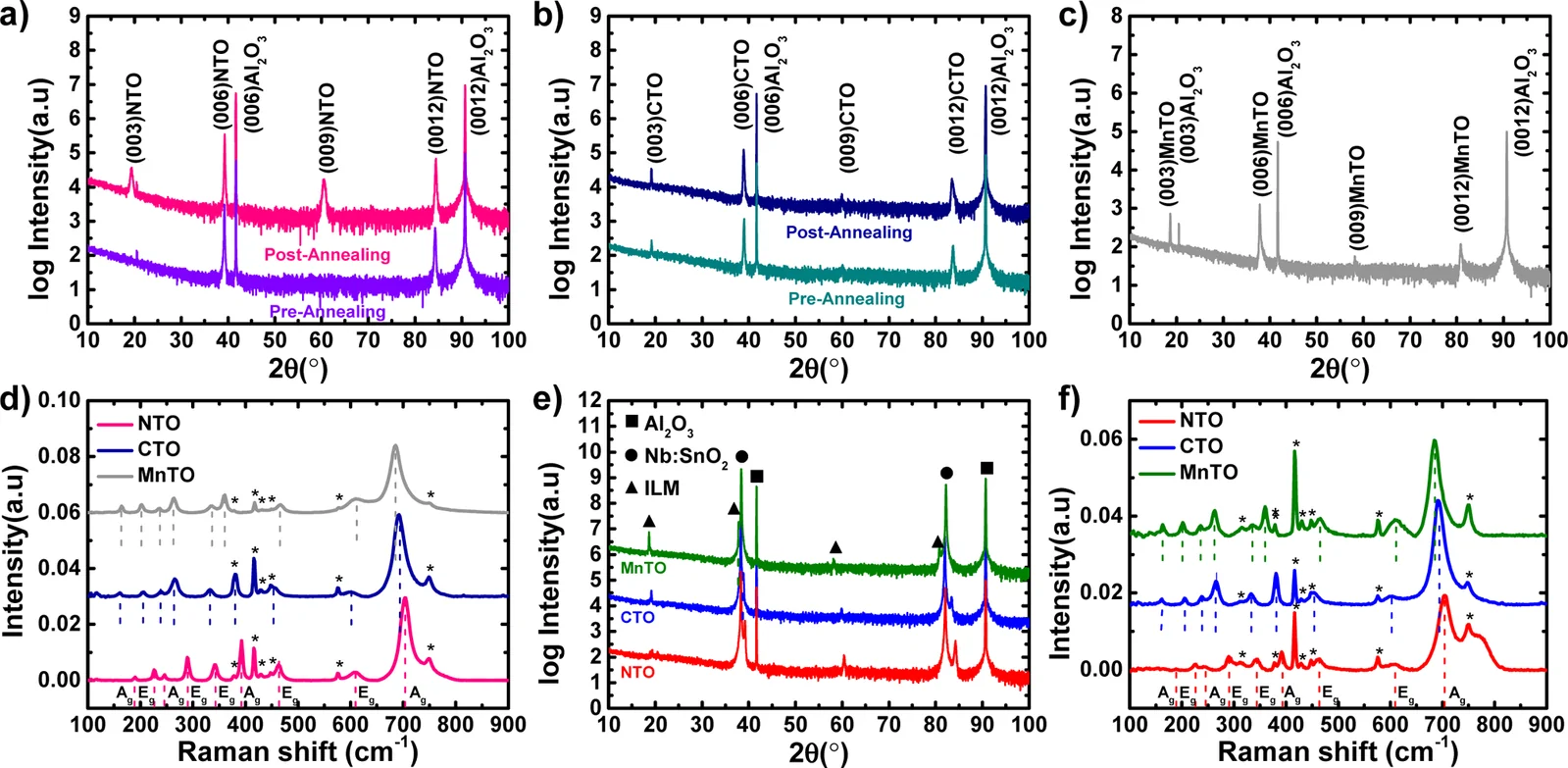
Structural Characterization of NTO, CTO, and MnTO on Al_2_O_3_(0001) and (100)­Nb:SnO_2_: (a) HR-XRD θ–2θ pattern for NTO grown on Al_2_O_3_(0001) showing the transition from space group *R*3̅*c* as deposited (purple) to *R*3̅ after annealing (pink); (b) HR-XRD θ–2θ pattern for CTO grown on Al_2_O_3_(0001) before and after annealing; (c) HR-XRD θ–2θ pattern for MnTO deposited at 1000 °C on Al_2_O_3_(0001); (d) phase characterization of pure-phase ilmenite thin films grown on Al_2_O_3_(0001) by Raman spectra; (e) HR-XRD θ–2θ patterns for the NTO, CTO, and MnTO films grown on (100)­Nb:SnO_2_; (f) Raman spectra of the NTO, CTO, and MnTO films grown on (100)­Nb:SnO_2_. In (d) and (f), the dashed lines are the vibrational modes for each ilmenite structure. Peaks marked with an asterisk (*) represent substrate peaks.

For HR-XRD analysis of the preannealed and postannealed CTO thin film, a similar procedure was applied as for NTO, using the same deposition conditions and annealing treatment, as shown in Figure 1b. Several key differences from NTO were observed. CTO has slightly different lattice parameters, resulting in a higher mismatch with the sapphire substrate and therefore, slightly reduced crystallinity compared to NTO. This is evident in a few aspects. First, as shown in Figure S2a, the 6-fold symmetry in the φ scan is not completely eliminated after annealing, with small peaks still appearing at 60° rotations. Second, as seen in Figure S2b, the rocking curve of the CTO film shows only a single, broad diffuse scattering component, indicating less overall structural order.

Unlike NTO and CTO, MnTO behaves differently in terms of deposition parameters. Stable epitaxial MnTO was grown on (0001)­Al_2_O_3_ at 1000 °C, as can be seen in Figure 1c. Initially, the NTO deposition conditions were used but instead of achieving a pure MnTO phase, a tetragonal TiMn_2_O_4_ structure was formed. To obtain the desired MnTO phase, the deposition temperature was increased to 900 °C and then to 1000 °C. As shown in Figure S3, this adjustment reduced the volume of tetragonal phase at 900 °C, and by 1000 °C, a phase-pure MnTO film was achieved. This indicates that the higher lattice mismatch between the substrate and MnTO requires a higher deposition temperature to promote better ordering. Notably, the annealing process did not further improve the ordering of the MnTO film, emphasizing that the deposition temperature was the critical factor. Figure S2 shows the HR-XRD scans for the film that was deposited at 1000 °C. In Figure S2c, the off-axis φ scan of the (1 0 10) reflection shows a 6-fold symmetry, indicating the presence of twinning defects. Figure S2d displays the rocking curve, which consists of a broad diffuse scattering component with fwhm of 0.5°, reflecting a high mosaic spread and lower structural order.

The Raman analysis was performed to confirm the absence of secondary phases and to verify that all vibrational modes associated with the ilmenite structure are present in each of the three thin film compositions grown on Al_2_O_3_(0001). The ilmenite structure, which crystallizes in the *R*3̅ space group (C_3i_^2^ symmetry), is expected to exhibit ten Raman active modes (5A_g_ + 5E_g_).^24^ As shown in Figure 1d, the spectra of all films align well with the characteristic peaks of the ilmenite structure, marked by dashed lines. One A_g_ mode is not clearly resolved in all films, likely due to its low intensity or overlap with nearby peaks, as noted in previous studies.^25^

Growth on conductive substrates such as Nb:SnO_2_ introduces additional challenges compared with sapphire. SnO_2_ is a wide-band-gap transparent oxide with a tetragonal crystal structure (space group *P*4_2_/*mnm*).^26^ Its high optical transparency in the visible range and enhanced conductivity from Nb doping make it a suitable current collector for photoanodes in water-splitting applications. However, the structural mismatch between SnO_2_ and ilmenite oxides complicates epitaxial growth. On sapphire, SnO_2_ itself grows epitaxially with three rotational domains,^27^ already introducing complexity into the crystalline framework. An additional challenge is the strong tendency of TiO_2_, which shares the same crystal structure as SnO_2_, to nucleate preferentially during growth.^13^ This competition increases the difficulty of obtaining phase-pure epitaxial ilmenite films on Nb:SnO_2_ and is often manifested as apparent deviations in cation stoichiometry during compositional analysis. Together, these factors highlight the need for careful optimization of deposition conditions.

Due to the tendency for isostructural TiO_2_ nucleation on the Nb:SnO_2_ current collector, stoichiometry control in MTiO_3_ films was found to be more sensitive to deposition conditions than for films grown directly on sapphire. Phase stability in many oxide systems is governed by the growth window defined by the oxygen partial pressure (*P*) and substrate temperature (*T*_s_),^28^ while in PLD, plume propagation and cation transfer are additionally influenced by the substrate–target distance (*D*_ST_) and the background gas pressure.^29,30^ The growth window for epitaxial NTO films on Nb:SnO_2_ was therefore mapped by systematically varying *P* and *D*_ST_, yielding an optimal Ni/Ti ≈ 1 ratio at *P* = 75 mTorr and *D*_ST_ = 100 mm (Figure S4, with a detailed description of the optimization provided in the Supporting Information).

Based on the optimized deposition parameters identified for NTO, CTO films were deposited using the same conditions. X-ray photoelectron spectroscopy (XPS) analysis confirmed an optimal Co/Ti ≈ 1 ratio under these parameters, as shown in Figure S5. In contrast to the nonstoichiometric transfer in NTO and CTO, MnTO did not exhibit this difficulty, which simplified its deposition process considerably. Consequently, the MnTO film was successfully deposited at *T*_s_ of 800 °C under an O_2_ partial pressure of 10 mTorr. The deposition parameters also included a standard *D*_ST_ of 70 mm and a repetition rate of 3 Hz.

XPS analysis of the NTO sample reveals that the Ni 2p spectrum (Figure S5a) is dominated by the Ni^2+^ oxidation state, with a minor contribution from Ni^3+^. The Ti 2p region (Figure S5b) shows a clear Ti^4+^ oxidation state, with noticeable asymmetric broadening toward higher binding energies compared to a TiO_2_ reference. This broadening is characteristic of Ti in the NTO lattice and is not attributed to the presence of an additional oxidation state.^31^ The O 1s spectrum (Figure S5c) was fitted with a Gaussian peak, corresponding to the O 1s core level. For the CTO sample, the Co 2p spectrum (Figure S5d) displays two main peaks at 781.7 eV (Co 2p_3/2_) and 797.5 eV (Co 2p_1/2_), along with two satellite shakeup peaks. The spin–orbit splitting of 15.8 eV matches the typical value for Co^2+^, indicating a 2+ oxidation state for cobalt.^32^ The Ti 2p spectrum (Figure S5e) shows peaks at 459.2 and 465.0 eV, corresponding to Ti 2p_3/2_ and Ti 2p_1/2_, respectively. The peak separation of 5.8 eV confirms the Ti^4+^ state in CTO.^32^ Similar to the NTO sample, asymmetric broadening toward higher binding energies is also observed in the Ti 2p region of the CTO sample, further supporting its assignment to Ti^4+^ within the CTO lattice. Finally, the O 1s region in the CTO sample (Figure S5f) shows no significant deviations. The MnTO sample (Figure S5g) exhibits a more complex peak structure. The main Mn 2p_3/2_ feature shows multiplet splitting peaks centered at 641.4 and 640.3 eV. This splitting is characteristic of the Mn^2+^ state and arises from intra-atomic interactions, specifically the Coulomb interaction between the 2p core hole and the 3d valence electrons.^33^ A minor contribution from the Mn^3+^ state is also observed at 642.7 eV. The Ti 2p spectrum (Figure S5h) displays a similar asymmetric broadening toward higher binding energies as seen in the NTO and CTO samples, supporting the presence of the Ti^4+^ oxidation state. Finally, Figure S5i displays the O 1s spectrum of the MnTO sample.

Structural and phase characterizations were conducted using HR-XRD and Raman spectroscopy to assess epitaxial film quality and phase composition across the films. Figure 1e presents the HR-XRD θ–2θ patterns, which confirm that all three films, NTO, CTO, and MnTO, were grown epitaxially with the same out-of-plane orientation being ilmenite(0001)//Nb:SnO_2_(100)//Al_2_O_3_(0001). Figure 1f shows the Raman spectra of all three samples grown on (100)­Nb:SnO_2_, which further confirm the absence of secondary phases. As previously observed, all Raman modes associated with the ilmenite structure are present, with one A_g_ mode not fully resolved, likely due to low intensity or spectral overlap. Additionally, Table 1 summarizes the Raman modes of the ilmenite structure and the corresponding experimental values for each film, as shown in Figure 1f. These values align well with previously reported data for NTO,^24^ CTO,^24^ and MnTO.^34^

**Table 1:** Raman Peak Positions [cm^–1^] for the Ilmenite Thin-Film-Grown Samples

Raman mode	NTO [cm^–1^]	CTO [cm^–1^]	MnTO [cm^–1^]
A_g_	188	161	163
E_g_	226	205	202
A_g_	244	238	236
E_g_	290	265	262
E_g_	343	333	335
A_g_	392	381	359
E_g_	464	454	464
A_g_	-	-	-
E_g_	609	602	609
A_g_	705	692	685

Figure S6 presents a series of HR-XRD scans to evaluate the crystallinity and quality of the thin films. Parts a–c of Figure S6 show the off-axis φ scans for NTO, CTO, and MnTO, respectively. The substrate exhibits a clear 3-fold azimuthal symmetry, as expected for the corundum crystal structure. Interestingly, the φ scan of the Nb:SnO_2_ film reveals a 6-fold symmetry, which is unexpected for a tetragonal rutile structure. This implies that the Nb:SnO_2_ grows epitaxially on the (0001) sapphire substrate with three rotational domains, each rotated 60° in-plane with respect to one another, due to the 6-fold symmetry of the *c*-plane surface of sapphire, as reported in previous studies.^27^ As a result, the growth of the absorbing layer also follows this 6-fold symmetry, influenced by the epitaxial growth of the transparent conducting oxide (TCO) layer. The φ scans show results similar to those discussed in the previous section, where 6-fold symmetry was observed for films grown directly on sapphire. However, while in that case the 6-fold symmetry arose from the large lattice mismatch leading to twinning defects, here it originates from the three rotational domains of the Nb:SnO_2_ bottom layer, which induce similar domains and associated twinning defects in the films. Parts d–f of Figure S6 display the X-ray reflectivity (XRR) scans for NTO, CTO, and MnTO, respectively, which were used to determine that the film thickness is approximately 30 nm. Maintaining consistent thickness is crucial for comparable PEC performance in the next sections. Parts g–i of Figure S6 show grazing incidence XRD measurements for NTO, CTO, and MnTO, respectively, confirming that no additional phases or out-of-plane orientations are present on the surface. Finally, parts j and k of Figure S6 illustrate the rocking curves for NTO and CTO, showing a narrow fwhm, which indicates low mosaic spread and high structural order. The rocking curve for the MnTO (006) reflection could not be evaluated due to peak overlap with the substrate.

To establish the epitaxial relationships, reciprocal space maps (RSMs) of the symmetric (0 0 6) and asymmetric (1 0 10) reflections were acquired for all three films grown on sapphire, as shown in Figure 2. Parts a and b of Figure 2 show the RSMs for the NTO sample after annealing. Table 2 summarizes the lattice parameters, theoretical ICDD values, and the lattice mismatch calculated from the measured parameters of both the film and the substrate, as extracted from the RSM results. The lattice mismatch (α) was calculated using the standard formula: $\alpha =\frac{a_{\mathrm{sub}}-a_{\mathrm{film}}}{a_{\mathrm{sub}}}$ , where *a*_sub_ and *a*_film_ are the lattice parameters of substrate and film, respectively. Before annealing, the sample is elongated in-plane and compressed out-of-plane. After annealing, the lattice parameters relax toward the theoretical ICDD values of the bulk material.

**Figure 2:**
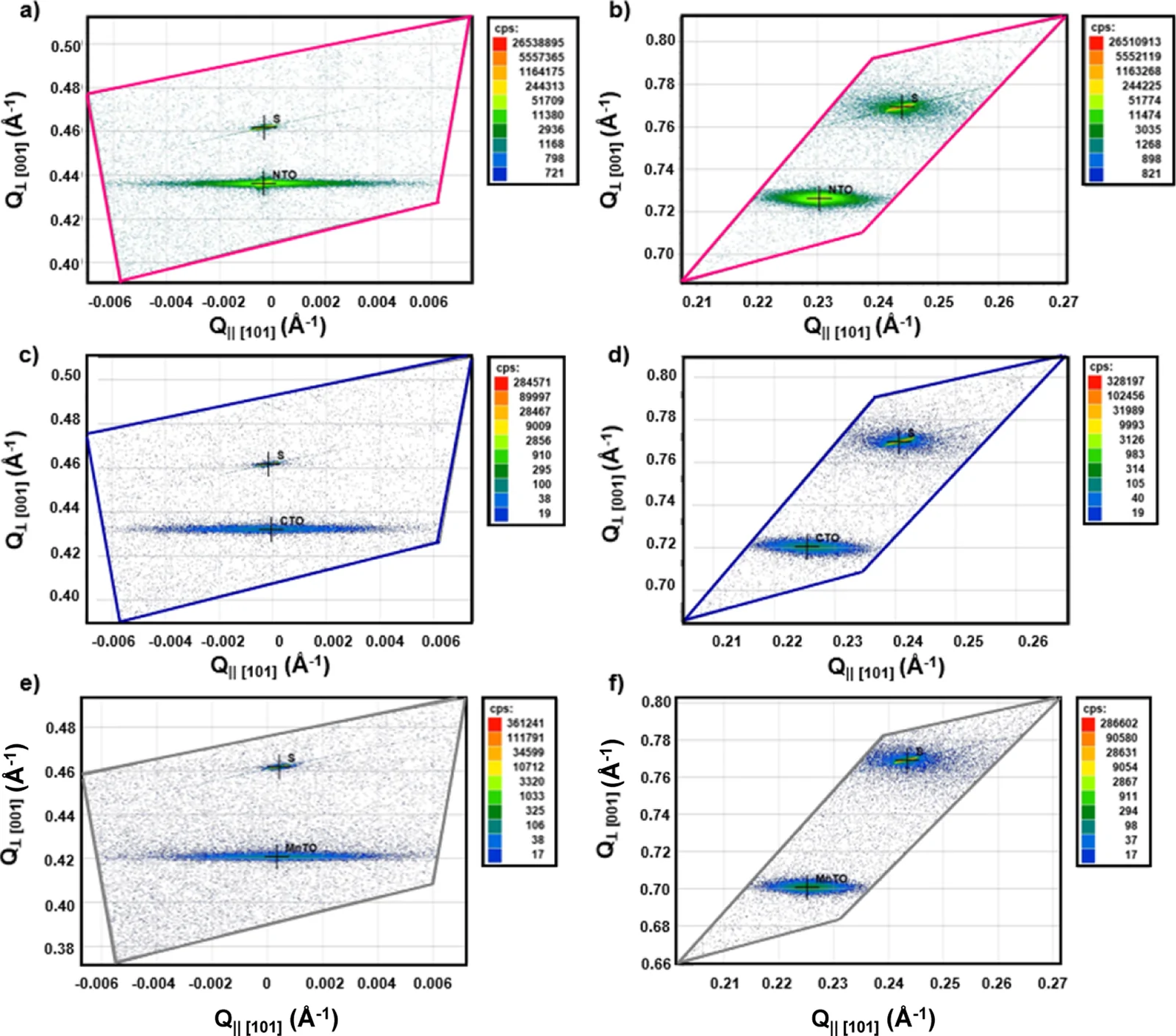
Reciprocal space maps of the symmetric (0 0 6) and asymmetric (1 0 10) reflections for the (a and b) NTO, (c and d) CTO, and (e and f) MnTO films grown on sapphire.

**Table 2:** Summary of the In-Plane and Out-of-Plane Lattice Parameters Derived from RSM for the Preannealed and Postannealed NTO and CTO Samples, along with the Corresponding Theoretical ICDD Values for the Materials

	ICDD NTO	preannealing NTO	postannealing NTO	ICDD CTO	preannealing CTO	postannealing CTO
*a* lattice parameter [Å]	5.030	5.27	5.02	5.0519	5.12	5.10
*a* mismatch [%]		–6.52	–5.8		–7.01	–6.58
*c* lattice parameter [Å]	13.790	13.64	13.76	13.9392	13.81	13.89
*c* mismatch [%]		–5.95	–5.8		–6.57	–6.88

Parts c and d of Figure 2 show the RSMs for the CTO sample after annealing. The lattice parameters of CTO undergo less relaxation toward the theoretical ICDD values after annealing compared to the NTO (as can be seen in Table 2), suggesting that higher temperatures or longer annealing times may be required. However, this was not pursued in order to maintain consistency in the experimental procedure. Finally, parts e and f of Figure 2 show the RSMs of the symmetric (0 0 6) reflection and the asymmetric (1 0 10) reflection for MnTO, respectively. These measurements indicate that the in-plane lattice parameter (*a*) is 5.15 Å, and the out-of-plane lattice parameter (*c*) is 14.26 Å, with mismatches of 8.3% and 9.75%, respectively. Compared to NTO and CTO, this higher mismatch helps explain why the crystallinity of the MnTO film is lower.

### 2.2 Optical Properties and Electronic Transitions

The optical properties of NTO, CTO, and MnTO films on sapphire are shown in Figure 3a, presenting the UV–vis spectra of the three samples. The displayed absorptance corresponds to the total film stack including the sapphire substrate. To extract the absorptance of the photoactive layers, the refractive indices (*n*) and extinction coefficients (*k*) of all three films were obtained from spectroscopic ellipsometry (SE) measurements and used in transfer matrix method (TMM) simulations. Figure 3b shows the extracted optical constants, with detailed ellipsometry modeling provided in Figure S7. Figure 3c presents a magnified view of the extinction coefficient (*k*) between 300 and 700 nm, highlighting differences in absorption features among the samples. The extracted optical constants were then used to calculate the specular reflectance via TMM simulations. Figure 3d compares the simulated and measured specular reflectance of NTO, with the remaining films shown in Figure S7. The good agreement between simulation and experiment validates the optical model, enabling reliable calculation of layer specific absorptance for all samples. The absorption features in the spectra can be correlated with specific electronic transitions in the films. Below 400 nm, the spectra primarily show charge-transfer transitions, likely from O^2–^ → Ti^4+^, as well as from O^2–^ → M^2+^ (M = Ni, Co, Mn), depending on the material. Above 400 nm, additional absorption features corresponding to LF transitions are observed, as shown in the magnified view of the spectra (Figure 3c) with the corresponding assignments labeled in Figure S8. The assignments, spectral positions, and selection rules are summarized in Table S1. For NTO, which consists of Ni^2+^ cations in octahedral coordination with a 3d^8^ electron configuration, the absorption around 450 nm can be attributed to overlapping ^3^A_2__g_(F) → ^3^T_1__g_(3P), ^3^A_2__g_(F) → ^1^A_1__g_(1G), and ^3^A_2__g_(F) → ^1^T_2__g_(1D) transitions.^14^ The first of these is spin-allowed, resulting in a stronger extinction coefficient and making it the most prominent feature in the spectrum. In CTO, the LF transition between 400 and 500 nm corresponds to the ^4^T_1__g_(F) → ^4^T_1__g_(P) transition of Co^2+^ in octahedral coordination with a 3d^7^ electron configuration.^15^ Additionally, the absorption band near 600 nm can be assigned to the ^4^A_2_(F) → ^4^T_1_(P) transition of Co^2+^ in a tetrahedral environment, suggesting small distortions in the structure that give rise to this feature.^16^ For MnTO, a broad absorption extending up to 660 nm is observed. This can be attributed to the Laporte forbidden and spin forbidden 3d^5^ multiplet transitions of Mn^2+^ in octahedral coordination. According to the Tanabe–Sugano diagram, this band corresponds to several LF transitions of the octahedral Mn^2+^ site, including ^6^A_1__g_(^6^S) → ^4^T_1__g_(^4^G), ^6^A_1__g_(^6^S) → ^4^T_2__g_(^4^G), ^6^A_1__g_(^6^S) → ^4^E_g_(^4^G), ^6^A_1__g_(^6^S) → ^4^A_1__g_(^4^G), ^6^A_1__g_(^6^S) → ^4^T_2__g_(^4^D), and ^6^A_1__g_(^6^S) → ^4^E_g_(^4^D).^17,18^

**Figure 3:**
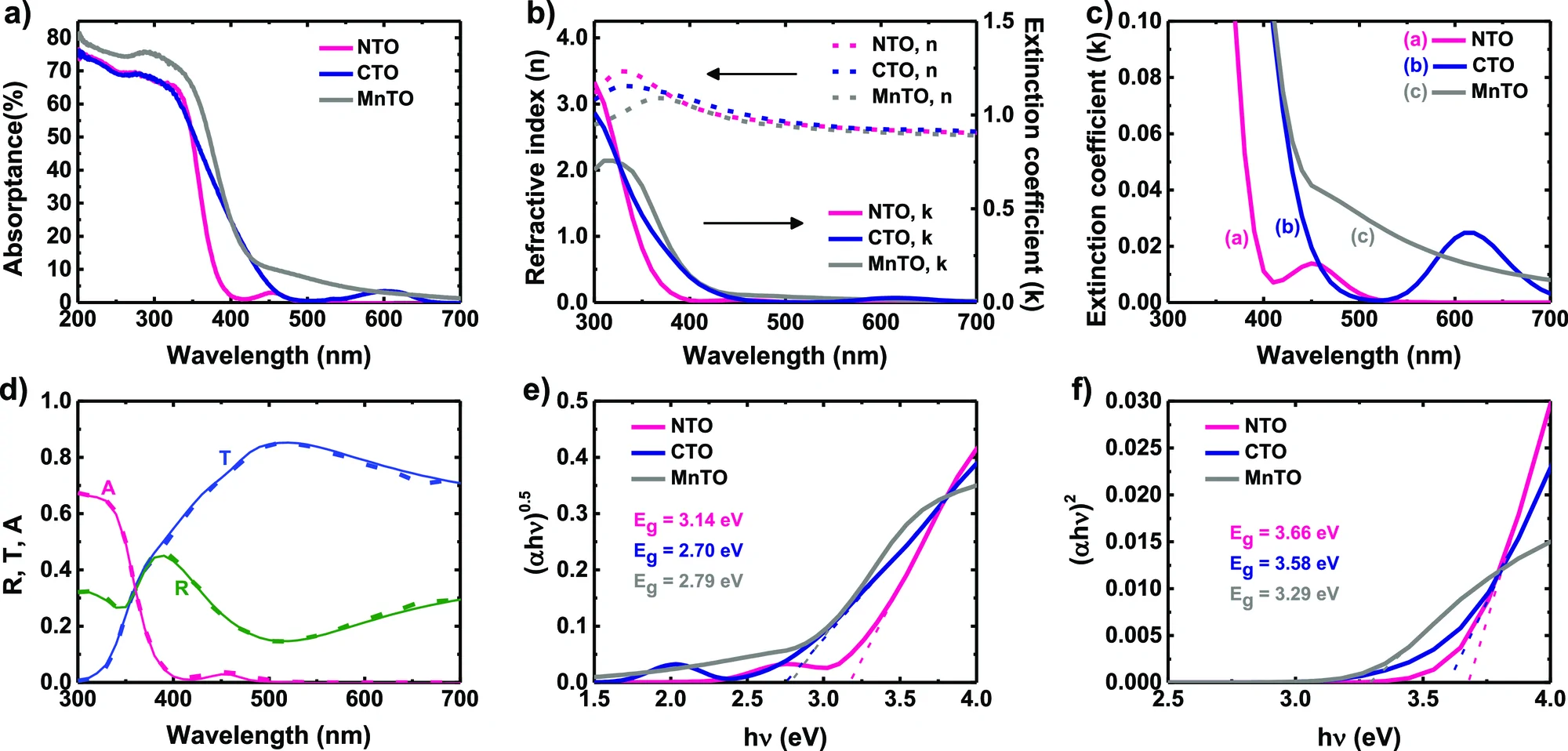
Optical characterization of the NTO, CTO, and MnTO thin films deposited on sapphire. (a) UV–vis absorptance spectra measured between 200 and 700 nm. (b) Optical constants extracted from SE for the three samples. (c) Zoomed-in view of the extinction coefficient (*k*) between 300 and 700 nm. (d) Simulated (dashed) and measured (solid) reflectance, transmittance, and absorptance of the NTO sample. Tauc plots used for (e) indirect-band-gap extrapolation and (f) direct-band-gap extrapolation from the ellipsometry measurements.

Parts e and f of Figure 3 show the Tauc plots for the indirect and direct band gaps of NTO, CTO, and MnTO, derived from the SE data. The plots reveal that MnTO has the smallest band gap, whereas NTO exhibits the largest, with the specific values indicated in the figure insets. This trend is consistent with the band gaps extracted from Tauc plots based on the UV–vis spectra of films grown on Nb:SnO_2_ TCO (Figure S9). Furthermore, it correlates with the theoretically absorbed photocurrent density (*J*_abs_), which follows the same order. From an absorption perspective, MnTO displays the highest potential for photocurrent generation, and NTO the lowest (Figure S9) under solar illumination. However, as discussed below, this trend does not translate into measurable mobile carrier yield or PEC performance.

### 2.3 PEC Performance

Figure 4 presents the PEC performance of the NTO, CTO, and MnTO films, measured in 1 M NaOH solution. In Figure 4a, linear-sweep voltammograms (LSVs) measured under chopped illumination are shown for potentials ranging from −0.4 to +1.7 V vs RHE. MnTO exhibits negligible photocurrent, as highlighted in the inset, where a small photoresponse with a minor reduction feature appears near 0.8 V vs RHE. Both NTO and CTO display ambipolar behavior. CTO shows a stronger photoanodic response, while NTO presents a slightly higher crossover potential, defined as the potential at which the photocurrent switches from cathodic to anodic behavior, together with a marginally enhanced photocathodic response. Figure 4b shows the photocurrent density (*J*_photo_ = *J*_light_ – *J*_dark_) confirming the trends observed in the chopped illumination. Figure 4c presents the EQE at 1.37 V vs RHE. Above 400 nm where LF transitions dominate, no significant photoresponse is observed for any of the films. Below 350 nm, NTO shows slightly higher efficiency, while between 350 and 400 nm, CTO performs marginally better. Although this difference is small, the higher photon flux in this spectral range under solar illumination contributes meaningfully to the photocurrent and is most likely the reason for the better photoanodic response of CTO under white light.

**Figure 4:**
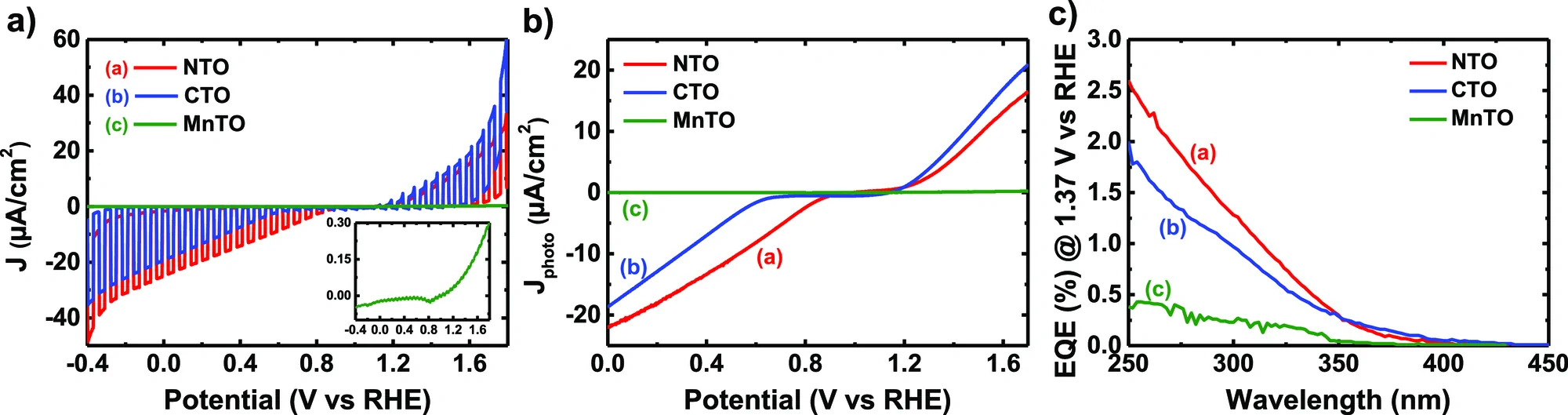
PEC characterization of the NTO, CTO, and MnTO thin films in 1 M NaOH solution. (a) LSVs under chopped illumination, (b) photocurrent density, and (c) EQE measurement at 1.37 V vs RHE.

To evaluate the influence of surface kinetics on the photoanodic response, measurements were conducted in 1 M NaOH containing 0.5 M Na_2_SO_3_ (Figure S10), where Na_2_SO_3_ serves as a hole scavenger to minimize surface recombination losses. While the hole scavenger increased the photocurrent for the NTO and CTO samples, the trend in the photoanodic region remained unchanged, indicating that differences in the photoanodic performance are primarily governed by bulk charge transport rather than surface recombination. Furthermore, chronoamperometry measurements under illumination were performed to confirm the stability of the photoelectrodes in the photoanodic region at 1.6 V vs RHE, as shown in Figure S11.

### 2.4 Charge-Carrier Dynamics Probed by TRMC

To further elucidate the dynamics of charge carriers that contribute to the observed photocurrent, we conducted TRMC measurements. TRMC monitors changes in reflected microwave power in response to a nanosecond laser pulse incident on the sample, revealing photoconductivity changes associated with generation and transport of mobile charge carriers. The TRMC signal is proportional to the product of the charge-carrier photogeneration yield and sum of the charge carrier’s mobility (ϕΣμ).^35^ In principle, TRMC detects all mobile charge carriers regardless of the underlying electronic transition, provided that the ϕΣμ product is above the noise floor and the carriers have not recombined within the instrumental response time. By making TRMC measurements as a function of wavelength, it may be possible to distinguish the effects of different transition types on photogenerated carrier behavior. However, in many transition-metal oxides such as hematite, this distinction is often challenging due to the spectral overlap of absorption bands.^9^ In the ilmenite system, the LMCT and LF transitions are more spectrally separated, with differences among the three compositions, as shown in the optical analysis. This separation allows us to correlate the wavelength-resolved TRMC signal with specific transitions and assess their relative contribution to the generation of mobile charge carriers.

Figure 5a compares the yield–mobility product (ϕΣμ) at an excitation wavelength of 310 nm for NTO, CTO, and MnTO, together with a comparison to an epitaxially grown rutile TiO_2_ thin film. TiO_2_, a d^0^ transition-metal oxide that lacks LF states and in which photocarrier generation arises from O^2–^ → Ti^4+^ LMCT transitions exclusively, provides a useful benchmark for efficient mobile carrier generation compared to the ilmenites, which contain partially filled d shells and therefore exhibit LF states. As expected, TiO_2_ exhibits the highest maximum yield-mobility product, ϕΣμ_max_, reflecting its superior TRMC response. MnTO shows a negligible response, while CTO demonstrates a higher ϕΣμ_max_ than NTO. The inset of Figure 5a magnifies the ilmenite-type samples for better comparison. Since TRMC measures the product ϕΣμ, the contributions of the photogeneration yield (ϕ) and the sum of the carrier mobilities (Σμ) cannot be decoupled directly; the higher ϕΣμ of CTO relative to NTO may therefore reflect a higher carrier yield, a higher mobility, or a combination of both.

**Figure 5:**
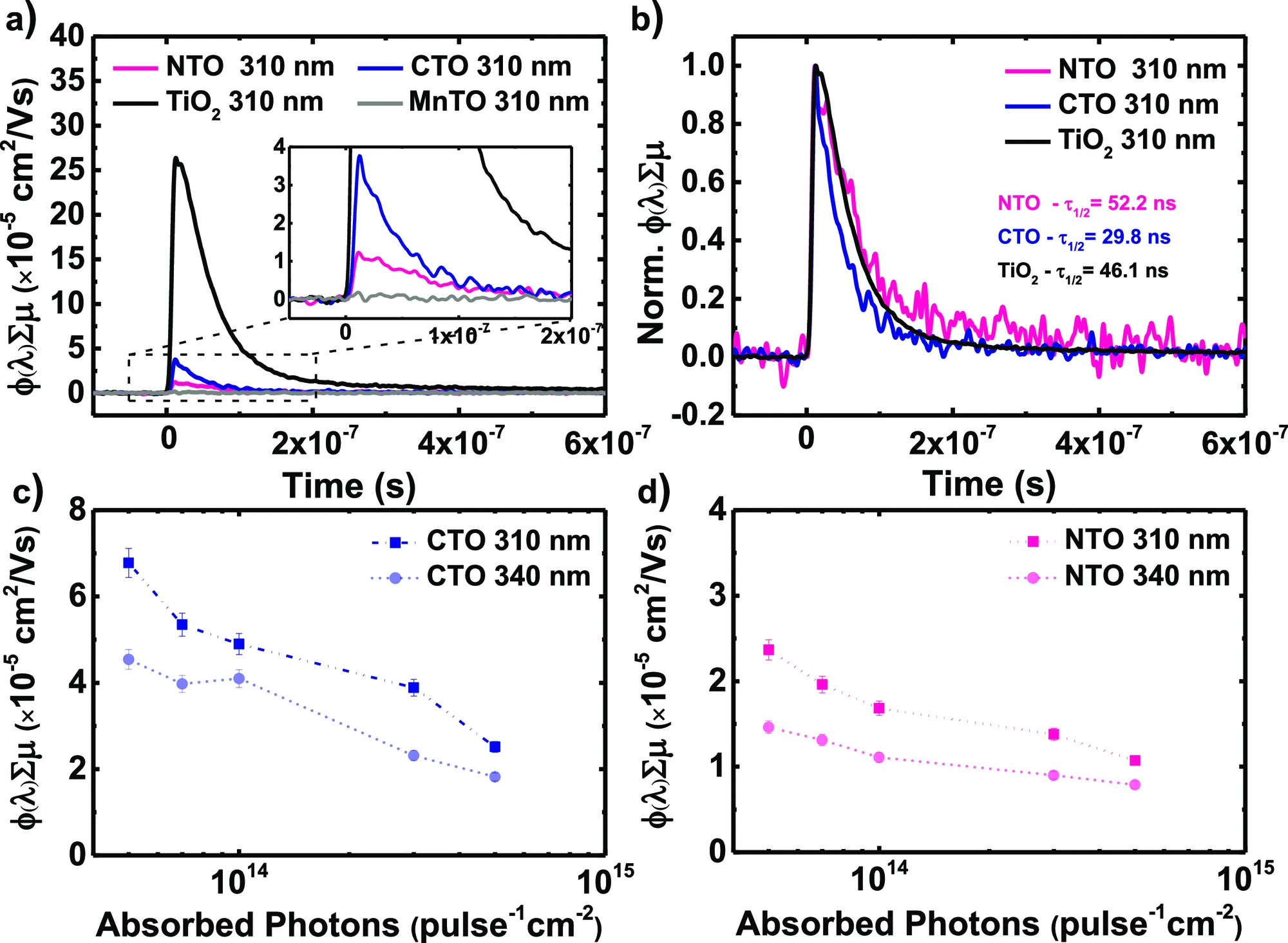
(a) TRMC transients measured at 310 nm at a photon fluence of 6 × 10^14^ photons pulse^–1^ cm^–2^ for TiO_2_, NTO, CTO, and MnTO films. (b) Normalized TRMC transients at 310 nm for TiO_2_, NTO, and CTO films. (c, d) ϕΣμ_max_ as a function of absorbed photon fluence at 310 and 340 nm for CTO and NTO, respectively.

Figure 5b compares the normalized yield–mobility product at 310 nm for NTO, CTO, and TiO_2_, allowing a direct comparison of their recombination dynamics. In the inset, the half-lifetimes of the photoconductance decays are provided for each film. It is evident that CTO exhibits the shortest half-lifetime, while NTO shows the longest. This indicates a tradeoff between the materials. Although CTO has a greater yield-mobility product, as seen in Figure 5a, NTO has longer carrier lifetime. Figure S12 shows complementary TRMC transient data for the CTO and NTO samples at different excitation wavelengths.

Parts c and d of Figure 5 show the peak TRMC photoconductivity of CTO and NTO, respectively, as a function of absorbed photon fluence at excitation wavelengths of 310 and 340 nm, selected as representative points within the UV charge-transfer absorption region. The normalized photoconductivity transients, shown in Figure S13, overlap well across the investigated excitation fluences, indicating similar temporal decay profiles. However, ϕΣμ_max_ decreases with increasing absorbed photon fluence at both 310 and 340 nm. Under purely first-order recombination, and assuming fluence-independent carrier mobility, ϕΣμ_max_ would remain approximately constant. The observed decrease therefore indicates that higher-order, carrier-density-dependent recombination processes become increasingly significant at elevated excitation densities.^36^ This behavior is observed at both wavelengths despite their differing absorption coefficients and hence carrier-generation profiles, indicating that the effect is carrier-density-dependent rather than specific to a single excitation condition.

Figure 6a presents the photoconductance action spectrum (PCAS), defined as the peak photoconductance normalized by the incident photon fluence, Δ*G*_max_/*I*_0_, measured by TRMC for the rutile TiO_2_ film at different excitation wavelengths (left *y*-axis), overlaid with its optical absorptance spectrum (right *y*-axis). The close correspondence between the PCAS and absorptance spectra indicates that the photogeneration yield of mobile carriers is effectively constant with respect to wavelength, meaning that absorbed photons above the band gap contribute similarly to the population of carriers detected by TRMC.^5^ This behavior is consistent with the d^0^ electronic configuration of TiO_2_, which lacks LF states and therefore does not provide competing localized excitation pathways. The origin of these contributing mobile charge carriers is attributed to O^2–^ → Ti^4+^ LMCT transitions.^37^ These results confirm TiO_2_’s high charge-carrier generation efficiency and are consistent with its well-established high IQE in PEC water splitting devices.^3^ The reduction of the PCAS relative to the absorptance at the shortest wavelengths is consistent with enhanced surface recombination, as the increasing absorption coefficient in the deep UV confines photocarrier generation progressively closer to the illuminated surface, rather than indicating a change in the character of the underlying optical transition.

**Figure 6:**
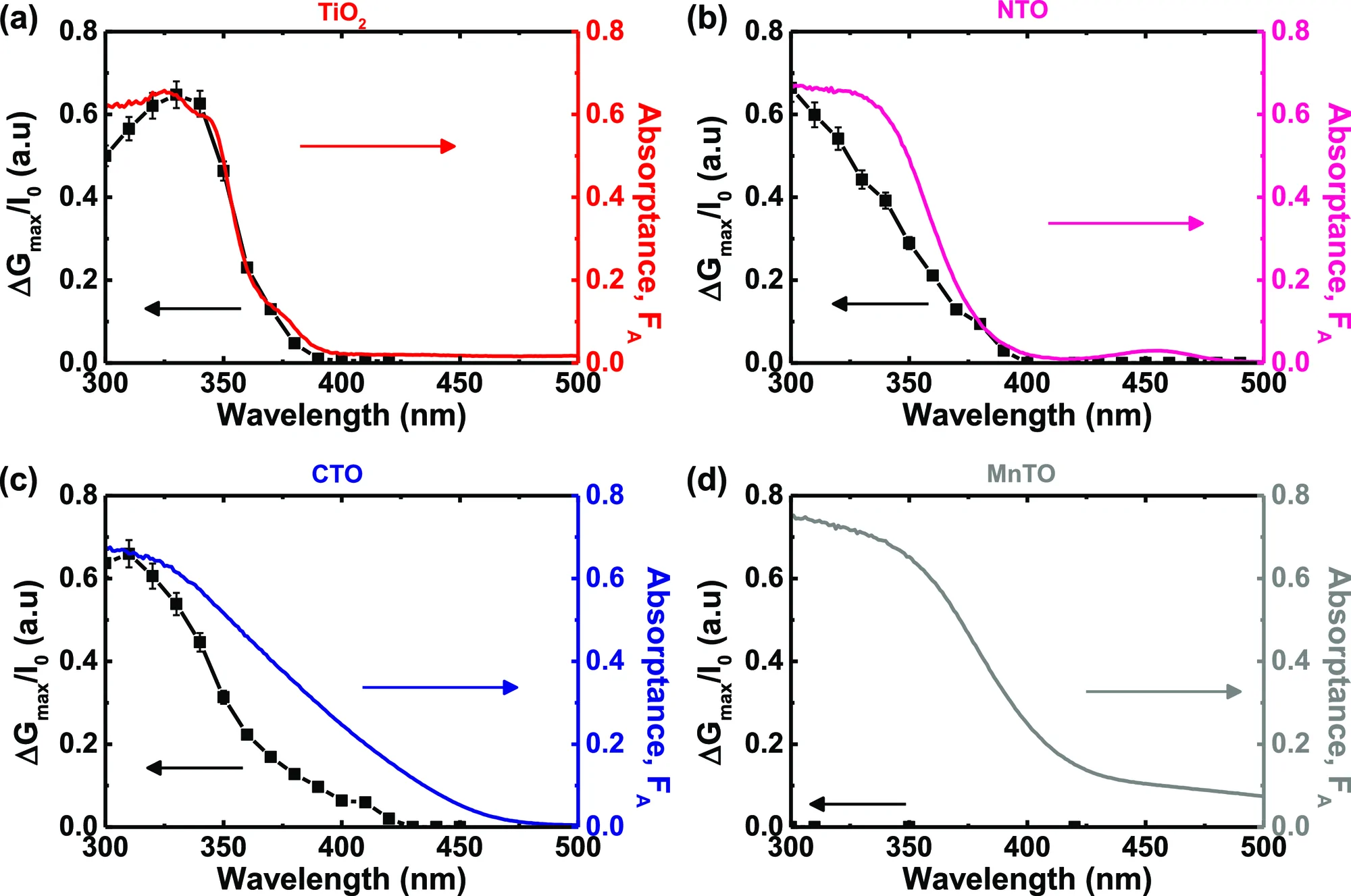
Peak photoconductance normalized by the incident photon fluence (Δ*G*_max_/*I*_0_, left *y*-axis), measured by TRMC at different wavelengths for (a) rutile TiO_2_, (b) NTO, (c) CTO, and (d) MnTO, scaled and overlaid with the absorptance spectrum of the corresponding film (right *y*-axis). No TRMC signal above the noise level was detected for MnTO at any excitation wavelength.

Parts b–d of Figure 6 show the PCAS spectra for NTO, CTO, and MnTO, each overlaid with the corresponding absorptance spectrum. The optical analysis indicated that MnTO exhibits numerous LF transitions that are both Laporte- and spin-forbidden and concentrated within the same spectral region. TRMC measurements further reveal the absence of any measurable photoconductivity. This observation is consistent with previous studies on Mn-based transition metal oxides, where photoexcited d-electrons were found to relax within only a few picoseconds, a time scale too short to yield measurable photoconductivity at the nanosecond resolution of TRMC.^38,39^ Taken together, these observations suggest that the dense manifold of spin-forbidden LF states in MnTO is associated with highly inefficient generation of mobile carriers, such that no measurable carrier population is detected on the nanosecond time scale of TRMC. This behavior contrasts with observations in hematite, where relaxation from charge-transfer states into the LF manifold was proposed to be spin-forbidden, partially mitigating these losses relative to other open d-shell oxides.^10^ The present results indicate that spin selection rules alone may not guarantee enhanced mobile carrier yield and that their impact likely depends on the energetic alignment and coupling between LF and charge-transfer manifolds, as well as the density of competing relaxation pathways. Further work is required to clarify how these factors jointly govern carrier yield across different open d-shell systems.

For NTO, the PCAS does not follow the absorption spectrum at short wavelengths. Although absorption is strong below ∼330 nm, the photoconductivity decreases sharply in this region. One possible explanation is that, in the deep-UV, the O^2–^ → Ti^4+^ and O^2–^ → Ni^2+^ LMCT transitions overlap spectrally as previously described, and may generate carriers with different mobilities. If the carriers associated with the O^2–^ → Ni^2+^ possess lower mobility, their contribution could lower the TRMC response when both transitions are excited simultaneously. Because TRMC is sensitive to the product ϕΣμ, a reduction in mobility would suppress the PCAS even if the photogeneration yield remains relatively high. Alternatively, relaxation through LF states could reduce the population of mobile carriers in this spectral region. Consistent with this hypothesis, excitation at wavelengths greater than 400 nm, where absorption is assigned to LF transitions, did not yield any measurable photoconductivity (Figure S14a).

CTO shows a redshift of the PCAS spectrum relative to NTO, consistent with the larger redshift seen in the absorption spectrum. However, the agreement between absorption and photoconductivity is noticeably poorer than in NTO. This larger deviation can be attributed to the additional presence of LF transitions that overlap with the LMCT region, further suppressing mobile carrier yield despite appreciable absorption. Above 430 nm, where LF transitions dominate, no photoconductivity is detected (Figure S14b), reinforcing the conclusion that such states do not generate measurable mobile carriers on the nanosecond time scales probed by TRMC.

To examine whether the spectral dependence of the PEC response reflects the intrinsic generation of mobile carriers rather than the depth at which light is absorbed, the EQE was normalized by the measured absorptance of the corresponding film to yield the IQE. Figure 7 compares the IQE with the absorptance-normalized TRMC response, given by ϕΣμ_max_(λ), for NTO and CTO under front illumination. When ϕΣμ_max_(λ) is multiplied by a single wavelength-independent scale factor, it closely follows the IQE spectrum across the charge-transfer region: for CTO the agreement extends over the full 300–420 nm range, while for NTO the agreement holds over 300–350 nm, with the deviation at longer wavelengths likely reflecting the lower TRMC signal of NTO and its steeply decreasing absorptance near the band edge. TRMC probes mobile carriers throughout the film volume and does not require transport to, or collection at, an interface. The agreement therefore suggests that the wavelength dependence of the IQE is governed primarily by the mobile-carrier response measured by TRMC within the nanosecond window, rather than by absorption depth.

**Figure 7:**
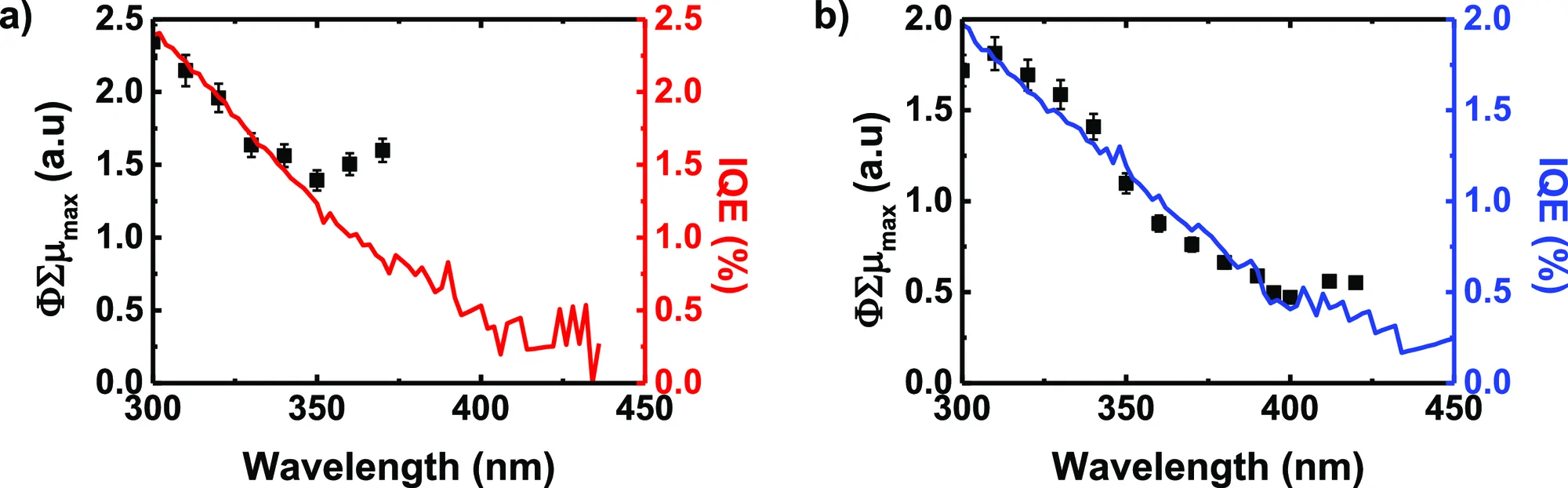
Comparison of the IQE (solid lines) with the absorptance-normalized TRMC response, ϕΣμ_max_(λ) (symbols), for (a) NTO and (b) CTO under front illumination. The ϕΣμ_max_(λ) spectrum was scaled by a wavelength-independent factor to plot the data on the same *y* scale as the IQE.

The IQE decreases with increasing wavelength under both front and back illumination (Figure S15) above 310 nm, even though the two geometries invert the carrier-generation profile within the film. If absorption depth were the dominant origin of the spectral decline, inversion of the generation profile would be expected to alter the wavelength dependence substantially. The persistence of the same decreasing trend above approximately 310 nm indicates that the wavelength dependence of the IQE is not dominated by the location of light absorption, but instead reflects the wavelength-dependent generation of mobile carriers. Below approximately 310 nm, the back-illumination IQE is progressively attenuated, attributed to parasitic absorption of deep-UV light in the SnO_2_-based current collector, which is not separated from the film absorptance in the measured stack.

Despite the differences between NTO and CTO discussed above, their overall wavelength-dependent photoconductivity trends remain similar and are concentrated in the UV region, as in TiO_2_. Previous ultrafast transient absorption spectroscopy studies on hematite and other open d-shell metal-oxides have shown that LF states can provide ultrafast relaxation pathways for carriers generated by charge-transfer excitation.^10^ The present TRMC measurements address the complementary case of direct LF excitation and show that excitation in the LF-dominated spectral regions does not produce measurable mobile carriers within the nanosecond measurement window. Importantly, the low apparent carrier yields under charge-transfer excitation persist despite the greater spectral separation between the LF and charge-transfer absorption bands in the ilmenites relative to hematite. This suggests that spectral separation alone does not prevent charge-transfer-generated carriers from subsequently relaxing into lower-lying LF states.

As noted above, TRMC alone cannot separate the carrier-generation yield from the summed mobility. However, a rough estimate of these relaxation losses can be obtained by taking the near-unity carrier-generation yield of TiO_2_ as a benchmark and assuming comparable summed carrier mobilities across the compounds. Under these assumptions, the ratio of ϕΣμ_max_ of each ilmenite to that of TiO_2_ approximates the fraction of absorbed photons that generate mobile carriers. In the 300–370 nm charge-transfer region, this procedure yields apparent mobile-carrier generation yields of approximately 3–6% for NTO and 6–15% for CTO; that is, roughly 94–97% and 85–94% of the absorbed photons fail to generate detectable mobile carriers on the nanosecond time scale. Under direct LF excitation, no mobile-carrier response is detected above the instrumental noise floor. Since lower summed carrier mobilities in the ilmenites relative to TiO_2_ would imply correspondingly higher carrier yields, the estimated carrier-generation losses represent upper bounds. In the opposite limiting case, where the carrier-generation yields are assumed to be unaffected, the same ratios would correspond to effective reductions in the summed carrier mobility by factors of approximately 17–35 for NTO and 7–17 for CTO.

Thus, together with the agreement between the IQE and ϕΣμ_max_(λ), the results indicate that UV charge-transfer excitations, rather than LF excitations, are responsible for the mobile carriers detected by TRMC and for the spectral PEC response of NTO and CTO. Taken together, the reported Ti 3d character of the low-lying unoccupied states^19^ and the broadly comparable nanosecond-scale TRMC decay kinetics of NTO, CTO, and TiO_2_ (Figure 5b) support O^2–^ → Ti^4+^ LMCT as the likely dominant contributor to mobile charge-carrier generation. However, the spectral overlap of the O^2–^ → Ti^4+^ LMCT, O^2–^ → M^2+^ LMCT, and intermetallic M 3d → Ti 3d charge-transfer excitations precludes quantitative separation of these channels using the present measurements. Indeed, Ti 2p-edge resonant inelastic X-ray scattering (RIXS) measurements on MTiO_3_ single crystals identified an intermetallic charge-transfer excitation at approximately 4–4.5 eV for M = Mn, Fe, Co, and Ni,^40^ placing it within the deep-UV charge-transfer manifold rather than the visible LF absorption region, although its optical oscillator strength, expected to be lower than that of the dipole-allowed LMCT transitions, is not established by RIXS.

## 3 Conclusions

Epitaxial ilmenite-type MTiO_3_ (M = Ni, Co, Mn) thin films were studied to elucidate the role of electronic excitations in photocarrier generation in open d-shell transition-metal oxides. Phase-pure, epitaxial films were obtained on sapphire and Nb:SnO_2_ substrates through optimization of PLD conditions. The MTiO_3_ films exhibit different optical absorption profiles, yet photocarrier yield depends on the type of electronic excitation rather than on absorption strength alone. Measurable photoconductivity is observed for NTO and CTO under UV excitation, consistent with UV charge-transfer transitions driving mobile charge-carrier generation. In contrast, MnTiO_3_ exhibits no detectable photoconductivity across the measured spectrum despite strong visible-light absorption. Absorption associated with LF transitions in all three oxides does not result in mobile charge carriers on the nanosecond time scales probed by TRMC and instead corresponds to localized excitations that relax without contributing to photoconductivity. These results indicate that charge-transfer transitions, with a likely major contribution from the O^2–^ → Ti^4+^ LMCT transition, are the dominant source of mobile charge carriers in ilmenite photoelectrodes, while LF excitations act as intrinsic loss channels in open d-shell oxides.

Based on these results, future work should focus on strategies that favor charge-transfer excitation and the rapid separation of the resulting carriers. Whether relaxation of charge-transfer-generated carriers through LF states can be mitigated remains an open question, but the present results provide useful guidance. The absence of a detectable response in MnTiO_3_, despite the spin-forbidden nature of its LF absorption, shows that this characteristic alone is insufficient to ensure a measurable mobile-carrier response. Likewise, the low ϕΣμ_max_ values of NiTiO_3_ and CoTiO_3_ under charge-transfer excitation, despite the clear spectral separation between their LF and charge-transfer absorption bands, indicate that spectral separation alone is insufficient to preserve a high mobile-carrier response. Although the respective contributions of carrier yield and mobility cannot be separated by TRMC alone, the suppressed response under charge-transfer excitation is consistent with relaxation of charge-transfer-generated carriers into lower-lying LF states. These observations suggest that the energetic alignment and electronic or vibronic coupling between the LF and charge-transfer manifolds, together with the rate of charge separation, may govern the competition between retention of mobile carriers and relaxation. Establishing how these factors interact, for example through advanced excited-state electronic-structure methods, is an important step toward developing mitigation strategies for open d-shell oxides.

## 4 Experimental Methods

### 4.1 Thin-Film Deposition

NTO, CTO, MnTO, and TiO_2_ custom targets were prepared by solid state reaction from pure NiO (99.995%), CoO (99.995%), MnO_2_ (99.995%), and TiO_2_ (99.9%) (NOAH Technologies Corp.) powders. The powders were mixed in appropriate proportions, and calcined for 6 h at 1100 °C, followed by uniaxial pressing and sintering at 1450, 1300, 1350 °C, and 1450 °C for 6 h, respectively. The target compositions were confirmed by XRF technique, and the crystalline structure was verified to be single phase by XRD. Thin films were deposited using a PLD system (PVD products, Nano-PLD1000). First, ∼200-nm-thick Nb:SnO_2_ layers were deposited onto *c*-plane (0001) cut sapphire substrates, serving as a TCO for PEC measurements. Subsequently, the absorbing layers of NTO, CTO, MnTO, and TiO_2_ were deposited on top. Before deposition, the vacuum chamber was pumped down to a base pressure lower than 5 × 10^–6^ Torr. Once the desired vacuum was attained, high-purity oxygen gas (99.999%) was released into the chamber at the desired partial pressure. The substrates were then heated to the desired set point temperature for the rest of the depositions. To provide a homogeneous film, substrates were rotated during film deposition at 1 rpm. The laser fluence was approximately 1.5 J cm^–2^ as measured with an energy meter. The laser repetition rate and the substrate to target distance were adjusted based on the specific material being deposited, as detailed in the results section.

### 4.2 XRD

HR-XRD studies were performed to examine the crystallinity, phase purity, and orientation of the films, using a Panalytical Empyrean III X-ray diffractometer (Cu Kα radiation), accompanied by a 2-bounce monochromator [Ge(220) channel cut], which was operated at 1.8 kW. Prior to the measurements, precise alignment steps were performed to adjust the height of the sample. High-resolution θ–2θ scans were performed to identify the out-of-plane crystallographic orientation of the films. Peaks were assigned with ICDD PDF-4 #00-033-0960 (NiTiO_3_), #04-016-5150 (CoTiO_3_), #04-012-0744 (MnTiO_3_), #01-072-7374 (TiO_2_), and #01-086-8560 (SnO_2_). In-plane alignment was examined by off-axis φ scans performed in skew-symmetric geometry. Grazing-incidence XRD with incident angles (ω) of 0.6°, 0.8°, and 1° was performed and a 2θ range between 10° and 70° was scanned with a step size of 0.05° step^–1^. XRR measurements were taken in parallel beam geometry, after which the XRR data was analyzed to extract the film thickness. Finally, RSMs of the symmetric and asymmetric reflections were obtained using 2-axis scans, which allowed the substrate and film peaks to be measured at the same ω offset.

### 4.3 Raman Analysis

Spectra were collected with a confocal Horiba LabRam HR evolution micro-Raman system, equipped with Syncerity Open Electrode CCD detector (deep-cooled to −60 °C, 1024 × 256 pixels). The light source was a 532 nm laser with a power of ∼30 mW on the sample. The laser was focused on the sample surface with MPlan N x100/0.9 objective (Olympus). Measurements were taken using a 100 μm confocal hole and 600 grooves/mm grating, with a spectral resolution of about 1.7 cm^–1^ and spot size of about 0.7 μm. An autocalibration procedure was done through Labspec software (Horiba, version 6.5.2) using a single crystal silicon (111) wafer. Spectra were taken in the range of 50–900 cm^–1^ with a 30 s exposure time, corrected for baseline by the Labspec software.

### 4.4 XPS

XPS measurements were performed in a Thermo Fisher Scientific ESCALAB Xi+ instrument. The work chamber pressure was ∼1 × 10^–10^ mbar. Photoelectron emission was achieved using an Al Kα (1486.6 eV) radiation source at room temperature, and an electron flood gun was used to minimize charging at the surface. High-resolution spectra for each element were measured using a step size of 0.05 eV and an average of at least five scans. Data were collected at 90° from the sample surface. The energy scale was shifted to adventitious carbon C 1s core-level line to fit the reference position of 284.8 eV.

### 4.5 Optical Measurements

The optical properties of the films were investigated using a combination of UV–vis spectroscopy and SE. UV–vis measurements were carried out with an Agilent Cary 5000 spectrophotometer in both reflection and transmission modes over the 200–800 nm range to determine the absorption characteristics of each photoanode. In parallel, SE was performed using a J.A. Woollam M-2000UI ellipsometer at incidence angles of 55°, 65°, and 75° across the 200–1000 nm range, and the data were analyzed with the CompleteEASE v.6.7 software.

### 4.6 PEC Characterization

PEC measurements of the samples were performed inside a “Cappuccino” electrochemical cell connected to a potentiostat (PalmSens4) in a three-electrode system with the sample as the working electrode, a pure Pt wire as the counter electrode, and Hg/HgO (1 M NaOH, pH = 13.6) as the reference electrode. LSVs in the dark and under light illumination (AM 1.5G, 100 mW cm^–2^) were obtained for the samples using an AA solar simulator (Abet Technologies model 10500) as the illumination source. EQE spectra were measured using a Sciencetech PTS-2 system from 250 to 500 nm in 2 nm increments, with the incident light chopped at 4 Hz and an illuminated aperture area of 0.1075 cm^2^. Measurements were performed at 1.37 V vs RHE in 1 M NaOH. The IQE was calculated by dividing the EQE by the measured absorptance at each wavelength.

### 4.7 TRMC

Optical excitation was provided by a wavelength-tunable optical parametric oscillator connected to a flash lamp-pumped Q-switched Nd:YAG laser (Ekspla NT342C), delivering ∼5 ns pulses at a frequency of 10 Hz. The TRMC setup utilized microwaves in the X-band range (8–12 GHz) for probing the sample, generated by a microwave signal generator (Keysight EXG N5173B). Microwave detection utilizes a zero-bias Schottky barrier diode (Fairview Microwave SMD0218). The signal is amplified using a high-speed amplifier (FEMTO HSA-X-1-40) with gain of 40 dB and bandwidth of 10 kHz to 1.2 GHz and channeled into a digital oscilloscope (Keysight EXR104A digital oscilloscope), triggered by the laser. The signal was averaged between 100 and 1000 times depending on the strength of the signal. The change in the microwave power reflected by the cavity after the laser pulse, Δ*P*/*P*, was monitored as a function of time, and correlated to the photoinduced change in the conductance of the sample, Δ*G*, by the following relationship:

$$\frac{\Delta P\left(t\right)}{P}=-K\Delta G\left(t\right)$$

where *K* is the sensitivity factor. From the experimentally observed change in the photoconductance (Δ*G*), the product of the charge-carrier generation yield [ϕ­(λ)] and the sum of electron and hole mobilities (∑μ) was calculated according to

$$\phi \left(\lambda \right)\sum \mu =\frac{\Delta G}{I_{0}\beta eF_{A}}$$

where *I*_0_ is the incident intensity per pulse, *e* is the elementary charge, β is the ratio between the inner broad and narrow dimensions of the waveguide, and *F*_A_ is the fraction of incident photons absorbed within the sample.

## Supplementary Material



## Notes

XRD patterns, and additional structural characterization of the MTiO_3_ films, growth-parameter optimization, XPS, optical absorption analysis, Tauc plots, and assignment of LF and charge-transfer transitions, TRMC excitation-fluence dependence and photoconductance transients, and IQE measurements under front- and back-side illumination (PDF)
