# Supplementary material for: Decoupling the Roles of Ligand-Field and Charge-Transfer Excitations on the Photocarrier Yield in Transition-Metal Oxides: Insights from MTiO3 (M = Ni, Co, Mn)
Source: Inorg Chem. 2026 Oct 8;65(41):24103–15. doi: 10.1021/acs.inorgchem.6c01924 (PMC13685420; doi:10.1021/acs.inorgchem.6c01924)
Supplement: Supplementary file 1 [file ic6c01924_si_001.pdf]

Supporting information

Decoupling the Roles of Ligand-Field and Charge-Transfer Excitations on Photocarrier Yield in Transition Metal Oxides: Insights from $MTiO_3$ (M = Ni, Co, Mn)

Ron Fishov[1], Yotam Engel[1], Rohit Kumar Saini[1], Sa'ar Shor Peled[1], Kumaraswamy Miriyala[1], Daniel A. Grave*[1,2]

[1]Department of Materials Engineering, Ben-Gurion University of the Negev, Beer Sheva 8410500, Israel
[2]Ilse Katz Institute for Nanoscale Science and Technology, Ben-Gurion University of the Negev, Beer Sheva 8410500, Israel
*Email: dgrave@bgu.ac.il

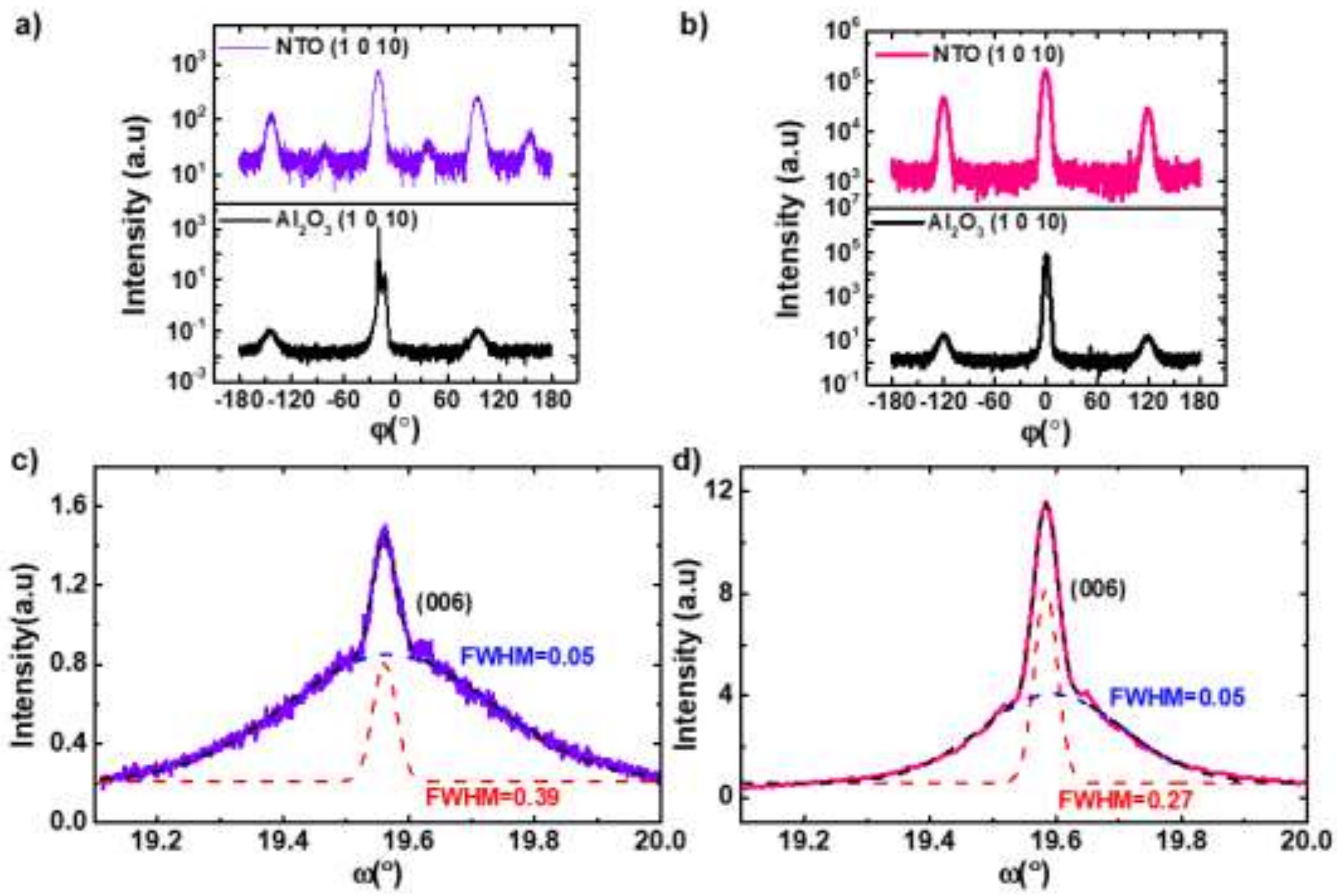

Figure S1: (a, b) off-axis phi scans of the (1 0 10) reflections from NTO and Al_2O_3 before and after annealing, respectively. Rocking curves deconvolution of the narrow specular component and the weak broad diffuse component for the (c) pre-annealed and (d) post-annealed samples of the NTO thin film;

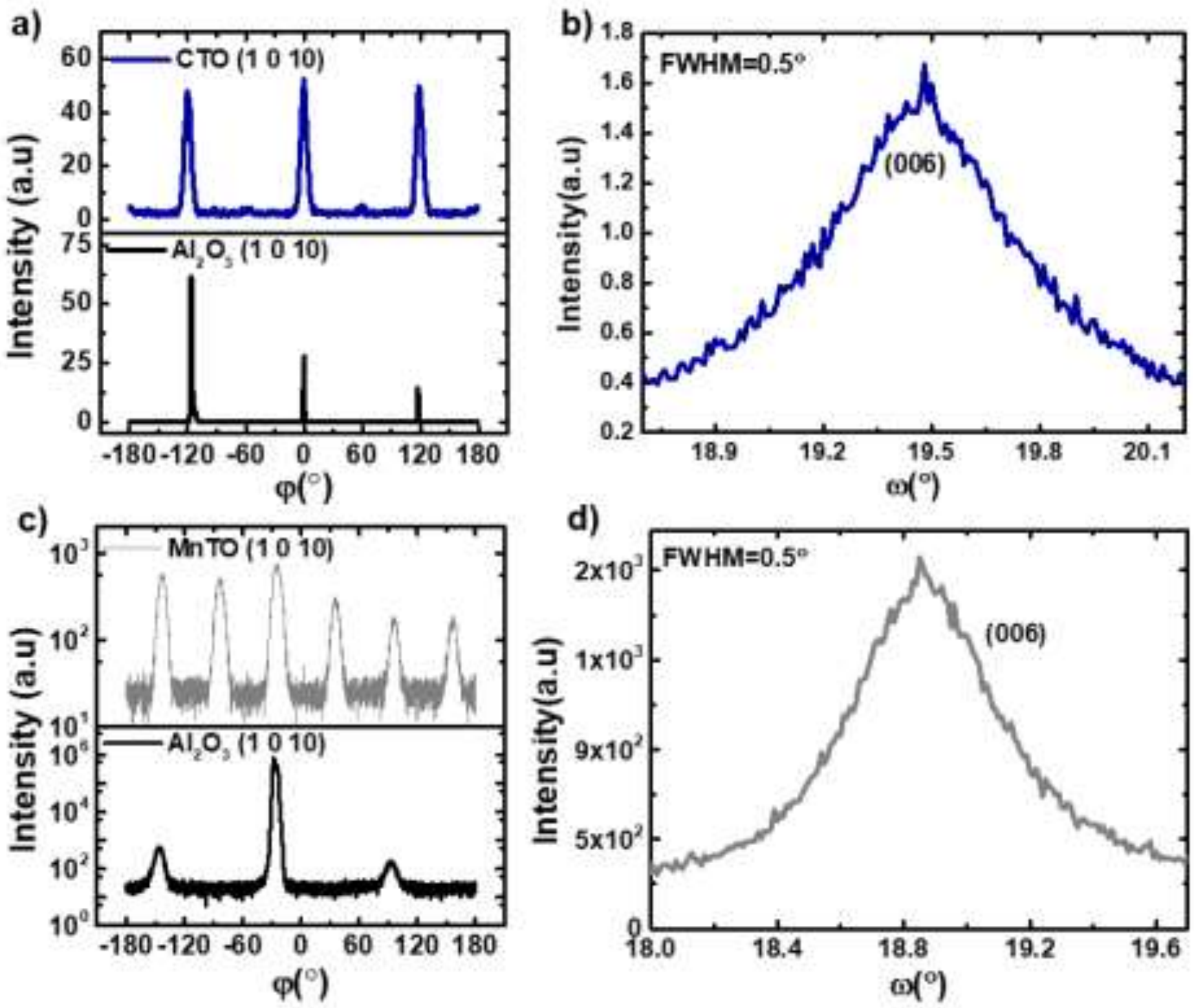

Figure S2: HR-XRD scans of the post annealing CTO sample on Al_2O_3(001) and MnTO deposited at 1000°C: (a) off-axis phi scan of the (1 0 10) reflections for CTO; (b) rocking curve of the (006) reflection of CTO; (c) off-axis phi scan of the (1 0 10) reflections for MnTO; (d) rocking curve of the (006) reflection of MnTO.

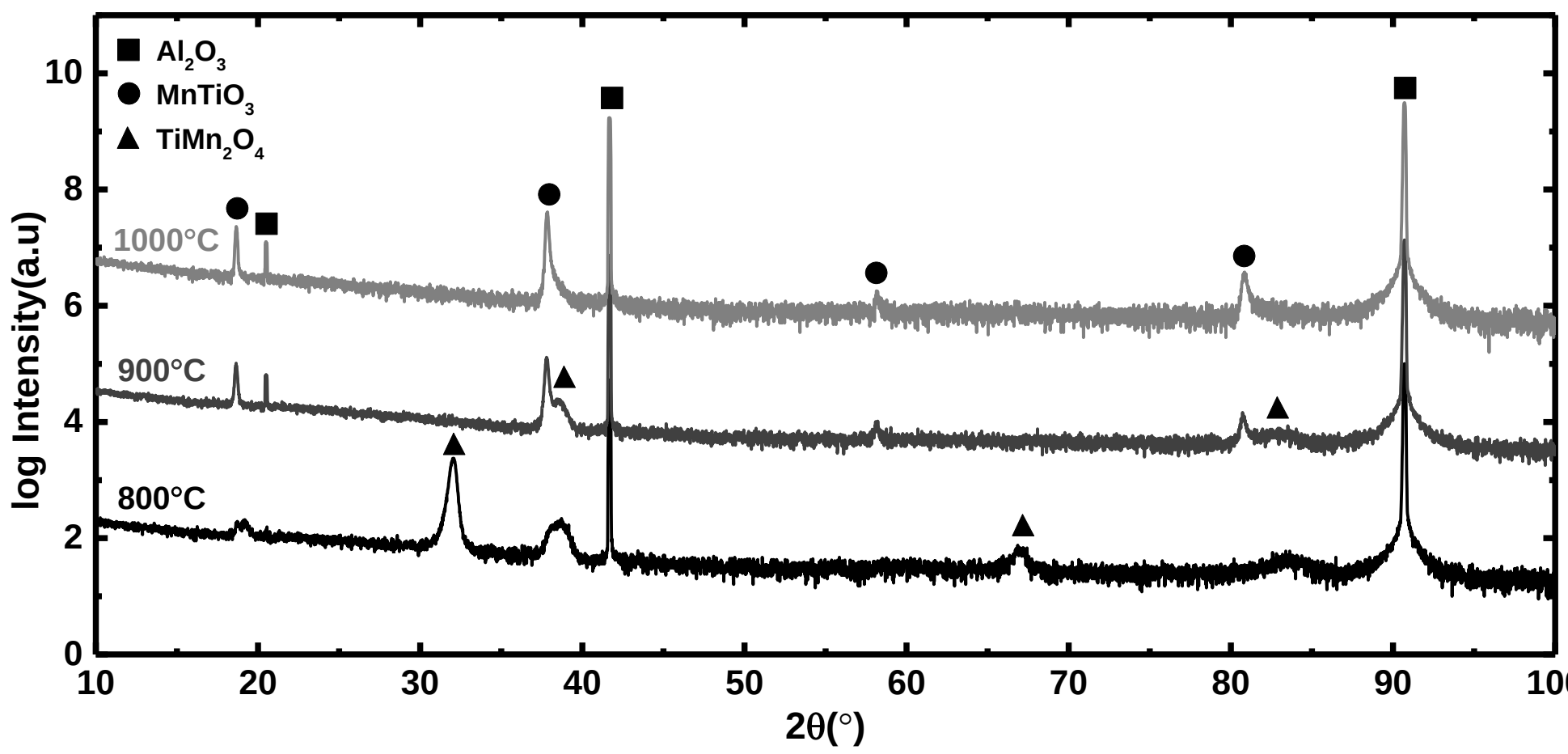

Figure S3: High-resolution θ–2θ XRD patterns for the films grown at 800°C, 900°C, and 1000°C.

To determine the growth window for epitaxial NTO films on Nb:SnO_2, the substrate temperature, laser fluence, and repetition rate were fixed at 700 °C, 1.5 J cm^{-2}, and 10 Hz, respectively, while the oxygen partial pressure (P) and substrate–target distance (D_{ST}) were systematically varied. Figure S4 illustrates the dependence of the film Ni/Ti ratio on P and D_{ST}, as determined by X-ray photoelectron spectroscopy (XPS). The oxygen pressure series is plotted in Figure S4a, and the substrate–target distance series in Figure S4b. For the oxygen pressure optimization, DST was held constant at 100 mm; for the DST optimization, P was fixed at 75 mTorr. The optimal Ni/Ti ≈ 1 ratio was obtained at P = 75 mTorr and DST = 100 mm.

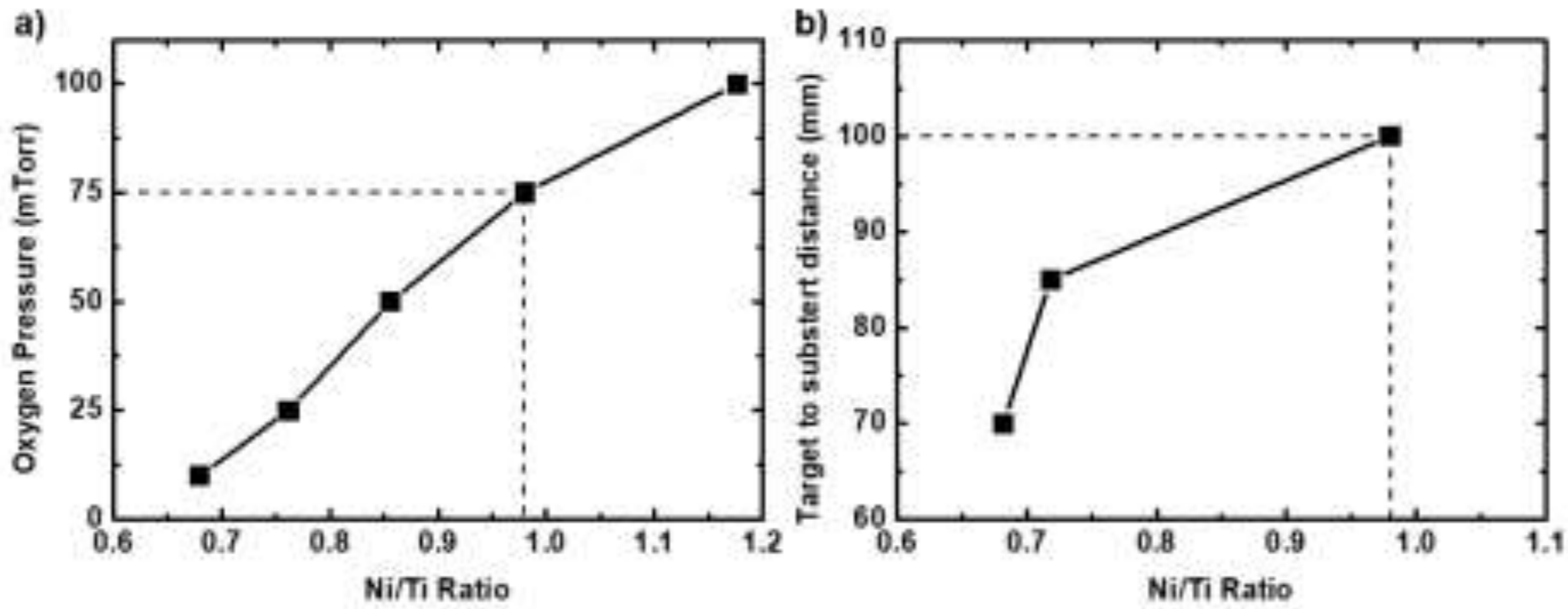

Figure S4: The dependence of the film Ni/Ti ratio on (a) the oxygen partial pressure P and (b) the substrate–target distance D_{ST}, for films deposited from a pure-phase NTO target.

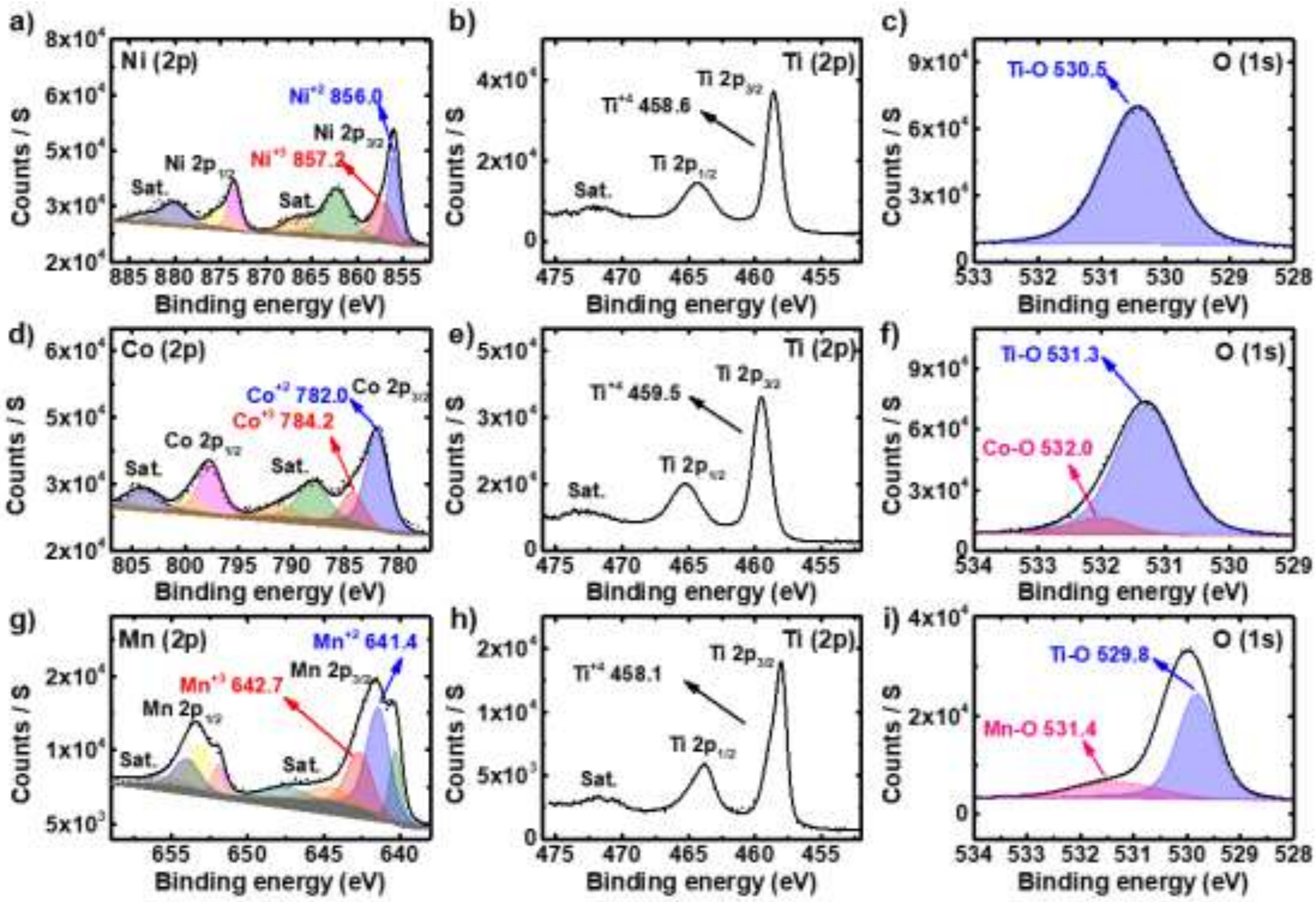

Figure S5: XPS spectra of the NTO film: (a) Ni 2p, (b) Ti 2p, and (c) O 1s; of the CTO film: (d) Co 2p, (e) Ti 2p, and (f) O 1s, and of the MnTO film: (g) Mn 2p, (h) Ti 2p, and (i) O 1s.

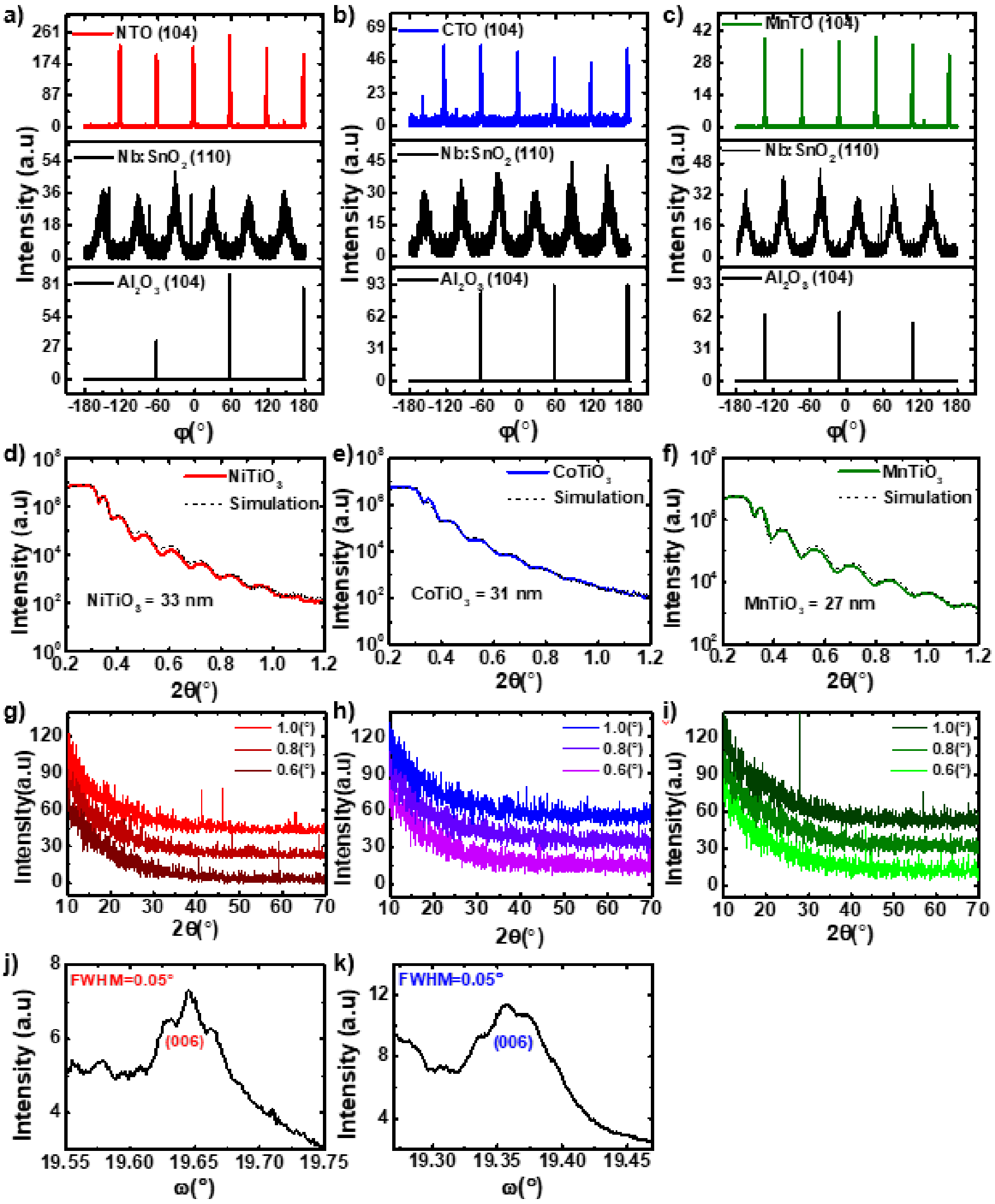

Figure S6: Structural analysis of the $NiTiO_3$, $CoTiO_3$, and $MnTiO_3$ thin film. (a-c) Off-axis phi scan for $NiTiO_3$, $CoTiO_3$, and $MnTiO_3$, respectively, (d-f) XRR of $NiTiO_3$, $CoTiO_3$, and $MnTiO_3$, respectively, (g-i) GIXRD scans with incident angles of 0.6 ˚,0.8 ˚, and 1.0˚ of $NiTiO_3$, $CoTiO_3$, and $MnTiO_3$, respectively, (j-k) Rocking curve of (006) plane for $NiTiO_3$ and $CoTiO_3$.

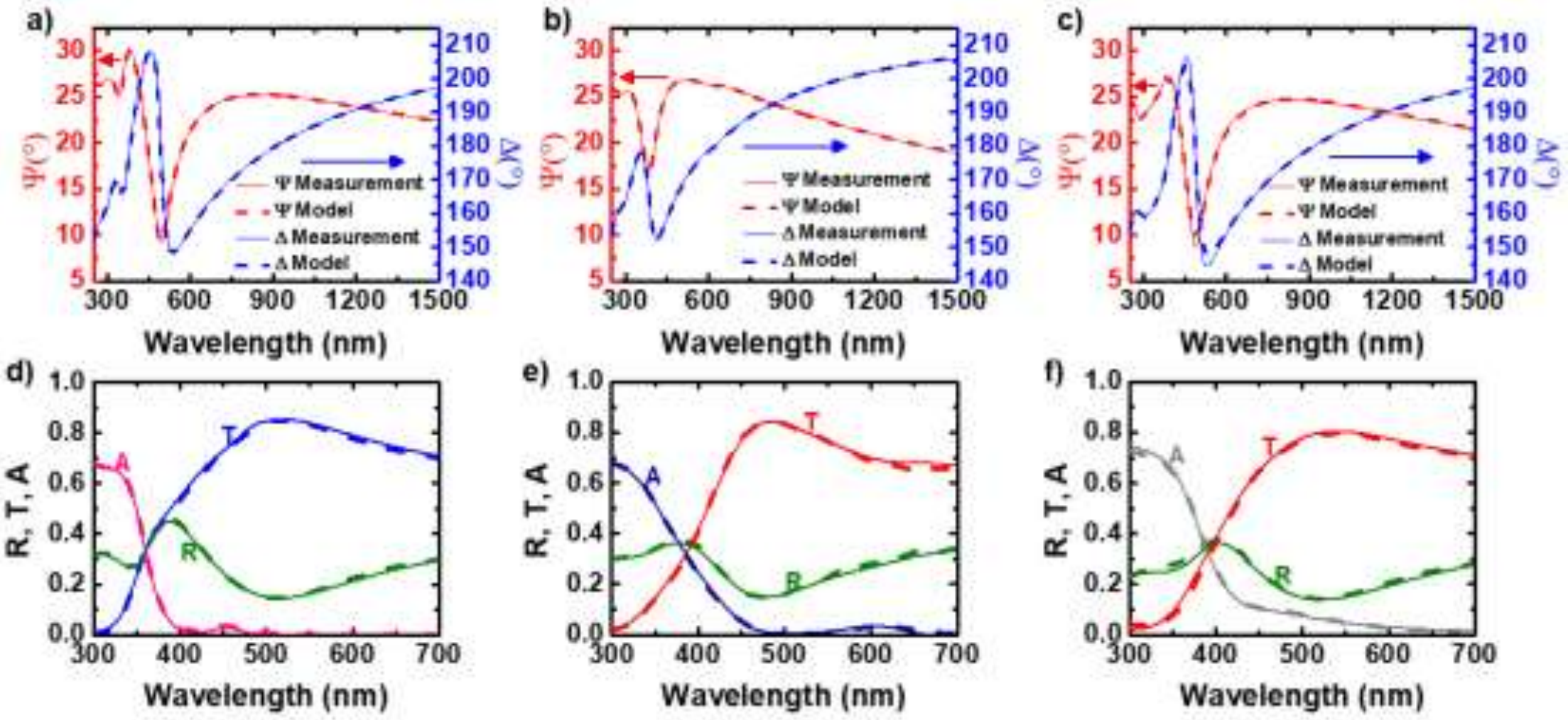

Figure S7: (a)-(c) Ellipsometry measurements and models of NTO, CTO, and MnTO, respectively. (d)-(f) Simulated and measured reflectance, transmittance, and absorptance of NTO, CTO, and MnTO, respectively.

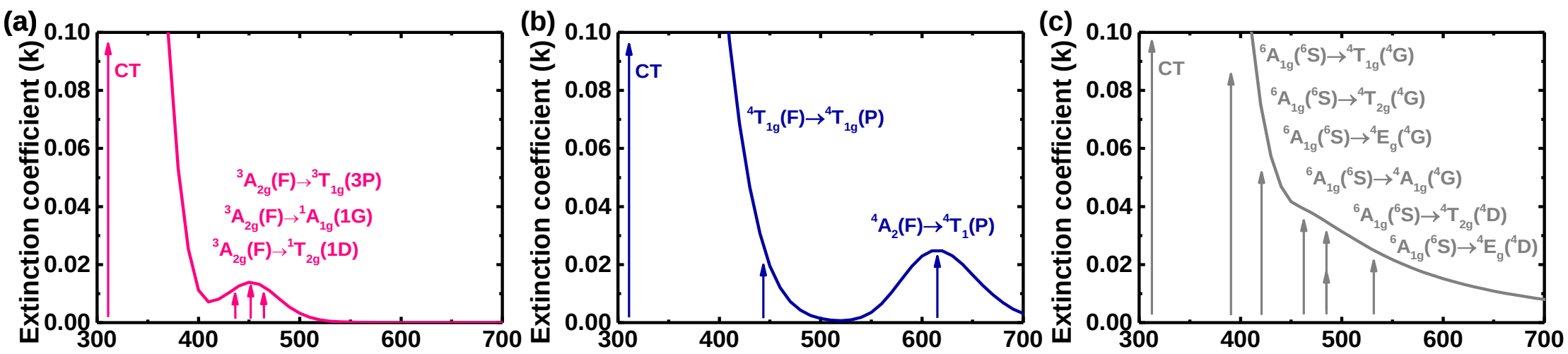

Figure S8: Zoomed-in view of the extinction coefficient (k) between 300-700 nm for (a) NTO, (b) CTO, and (c) MnTO with assignments according to references in Table S1.

Table S1: Assignment of the optical transitions observed in NTO, CTO, and MnTO thin films. Reference numbers correspond to the main-text bibliography.

Material	Spectral region	Transition	Type	Ref.
NTO, CTO, MnTO	< 400 nm	$O^{2-} \rightarrow Ti^{4+}$	LMCT	14,17,19
NTO, CTO, MnTO	< 400 nm	$O^{2-} \rightarrow M^{2+}$ (M = Ni, Co, Mn)	LMCT	14,19
NTO	~450 nm	$^3A_{2g}(F) \rightarrow {}^3T_{1g}(^3P)$, Ni^{2+} (O_h, d^8)	LF, spin-allowed	14
NTO	~450 nm – 510 nm	$^3A_{2g}(F) \rightarrow {}^1A_{1g}(^1G)$, Ni^{2+} (O_h, d^8)	LF, spin-forbidden	14
NTO	~450 nm – 510 nm	$^3A_{2g}(F) \rightarrow {}^1T_{2g}(^1D)$, Ni^{2+} (O_h, d^8)	LF, spin-forbidden	14
CTO	400–500 nm	$^4T_{1g}(F) \rightarrow {}^4T_{1g}(P)$, Co^{2+} (O_h, d^7)	LF, spin-allowed	15
CTO	~600 nm	$^4A_2(F) \rightarrow {}^4T_1(P)$, Co^{2+} (T_d)	LF, spin-allowed	15,16
MnTO	up to ~660 nm	$^6A_{1g}(^6S) \rightarrow {}^4T_{1g}(^4G)$, $^4T_{2g}(^4G)$, $^4E_g(^4G)$, $^4A_{1g}(^4G)$, $^4T_{2g}(^4D)$, $^4E_g(^4D)$, Mn^{2+} (O_h, d^5)	LF, spin- and Laporte-forbidden	17,18

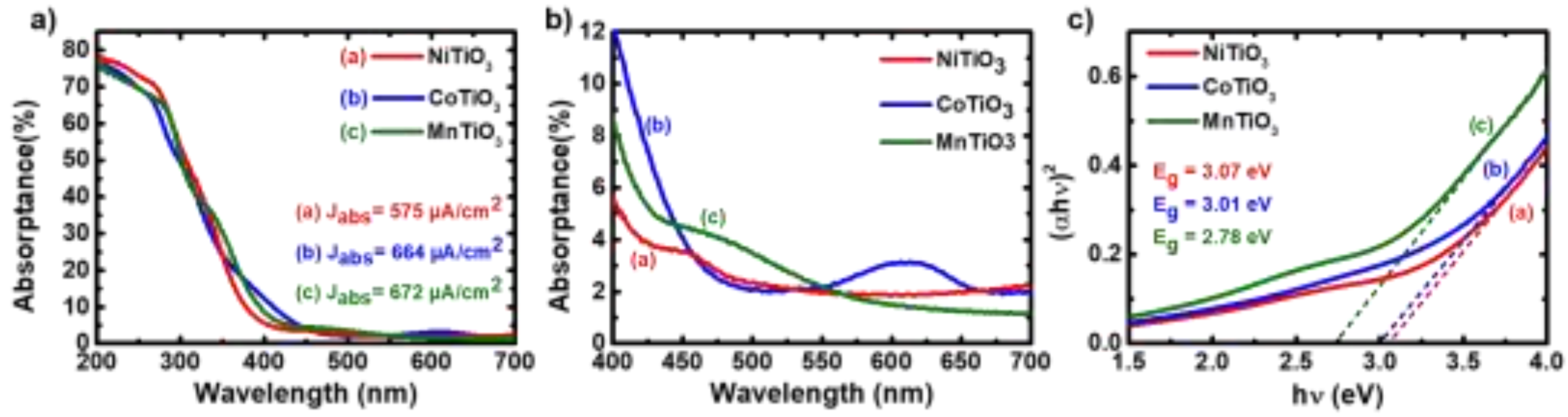

Figure S9: Optical characterization of the $NiTiO_3$, $CoTiO_3$, and $MnTiO_3$ thin films. (a) UV-vis absorptance spectra measured between 200-700 nm. (b) Magnified view of the visible range. (c) Tauc plots used for direct band gap extrapolation.

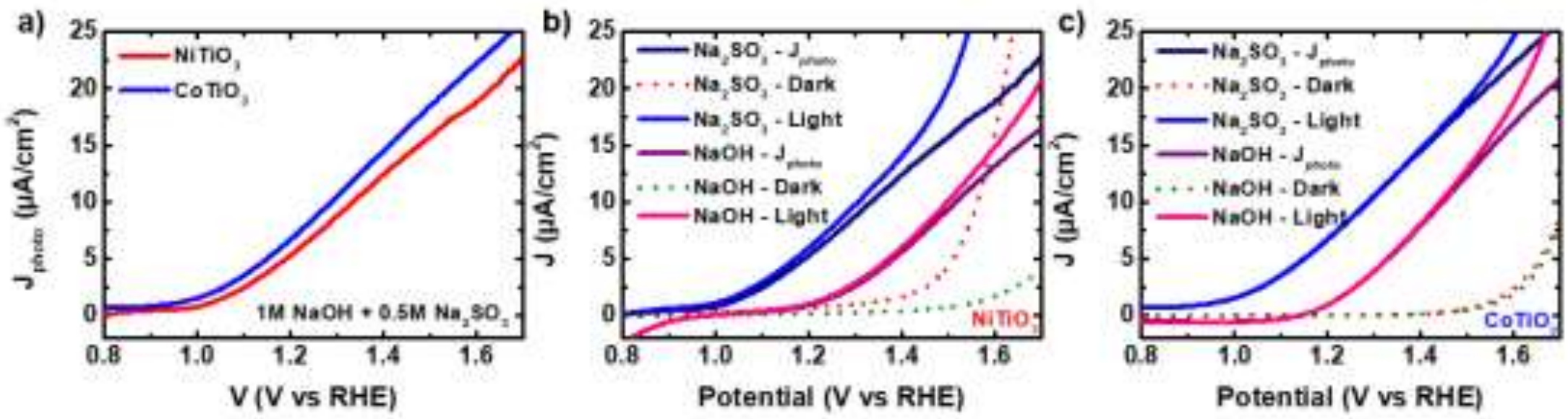

Figure S10: (a) Anodic photocurrent density in 1M NaOH + 0.5M Na_2SO_3 solution. Comparison of current density vs. Potential (V vs RHE) measured in 1M NaOH and 1M NaOH + 0.5M Na_2SO_3 hole scavenger for (b) NTO, and (c) CTO samples.

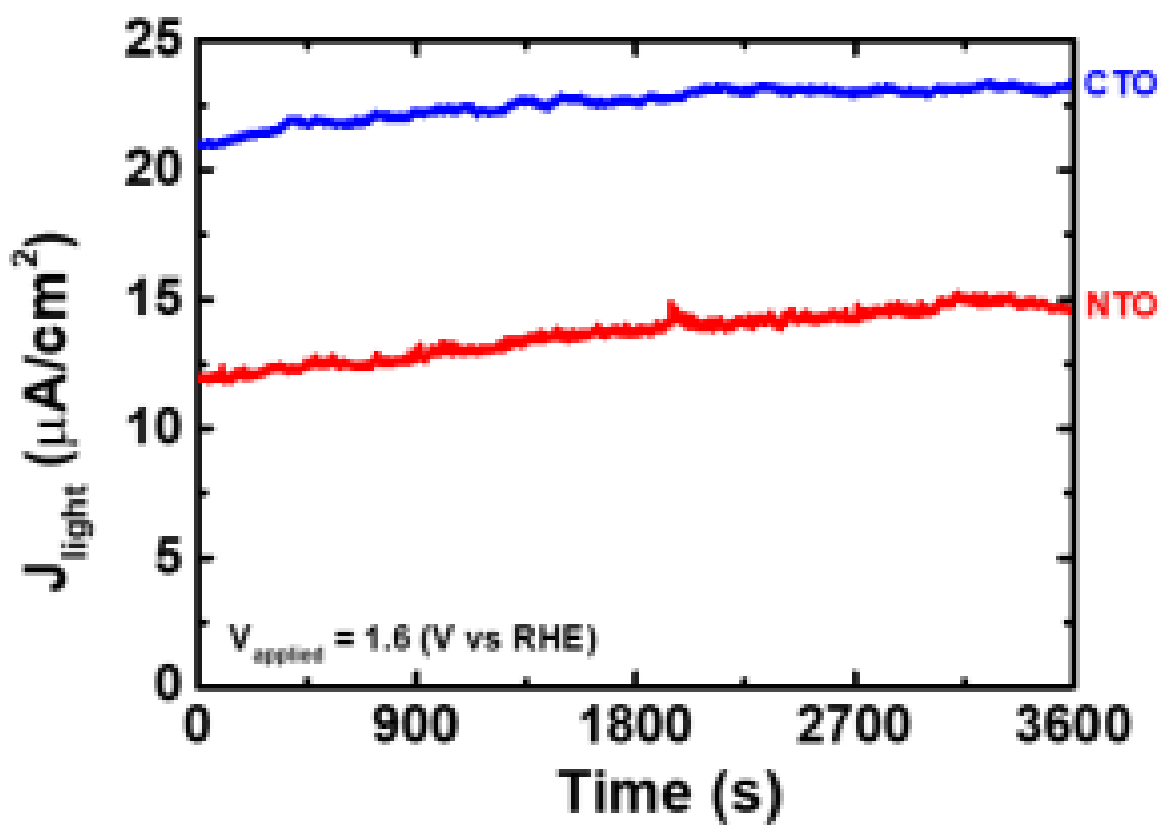

Figure S11: stability measurements by chronoamperometry at 1.6 V vs RHE.

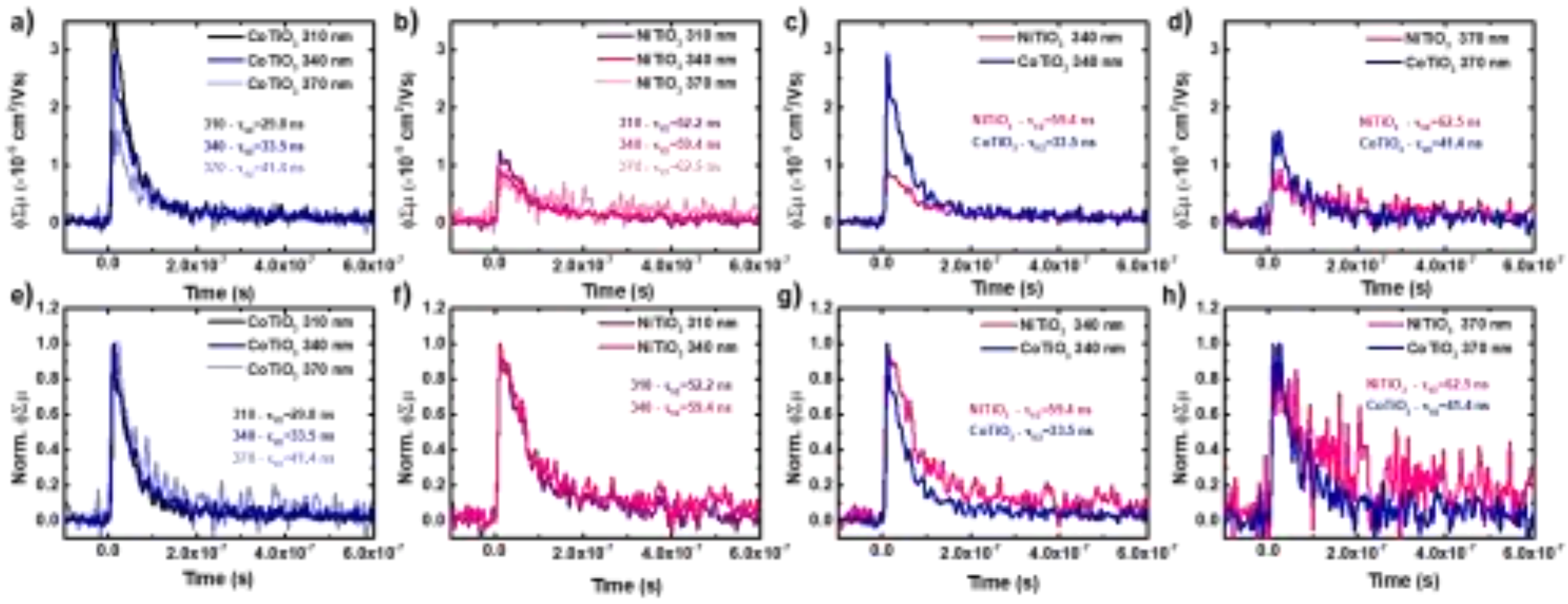

Figure S12: TRMC transients measured at 310 nm, 340 nm, and 370 nm excitation wavelengths at a photon fluence of $6x10^{14}$ $pulse^{-1}cm^{-2}$ for (a) CTO and (b) NTO films. Comparison of TRMC transients at (c) 340 nm, and (d) 370 nm between CTO, and NTO films at a photon fluence of $6x10^{14}$ $pulse^{-1}cm^{-2}$. Normalized TRMC transients measured at 310 nm, 340 nm, and 370 nm excitation wavelengths for (e) CTO and (f) NTO films. Comparison of normalized TRMC transients at (g) 340 nm, and (h) 370 nm between CTO, and NTO films.

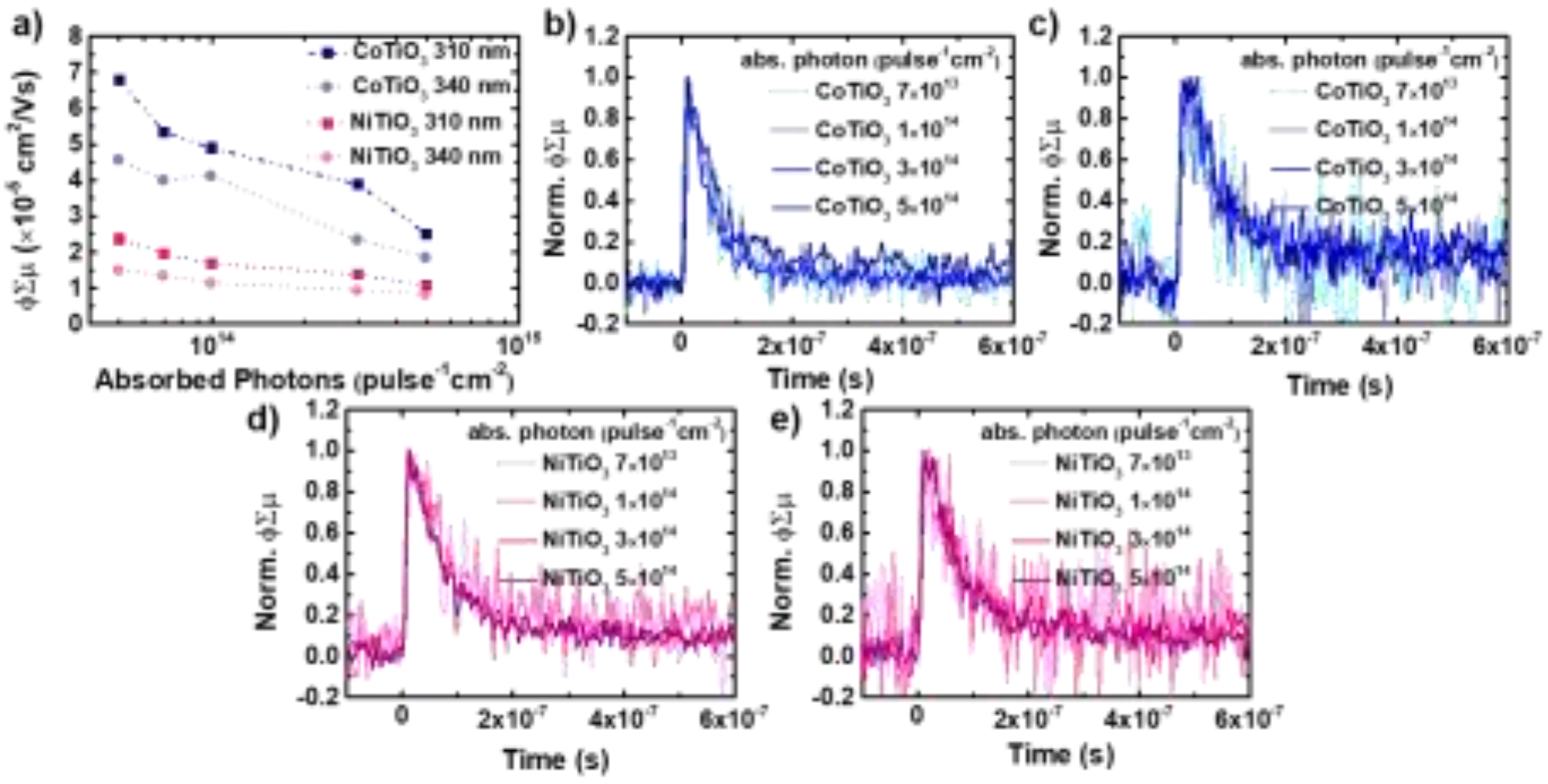

Figure S13: (a) $\phi\Sigma\mu_{max}$ as a function of absorbed photons per pulse and per cm^2 at 310 and 340 nm for CTO and NTO thin films. Normalized TRMC transients measured at (b) 310 nm, and (c) 340 nm excitation wavelengths across different intensities for the CTO film. Normalized TRMC transients measured at (d) 310 nm, and (e) 340 nm excitation wavelengths across different intensities for the NTO film.

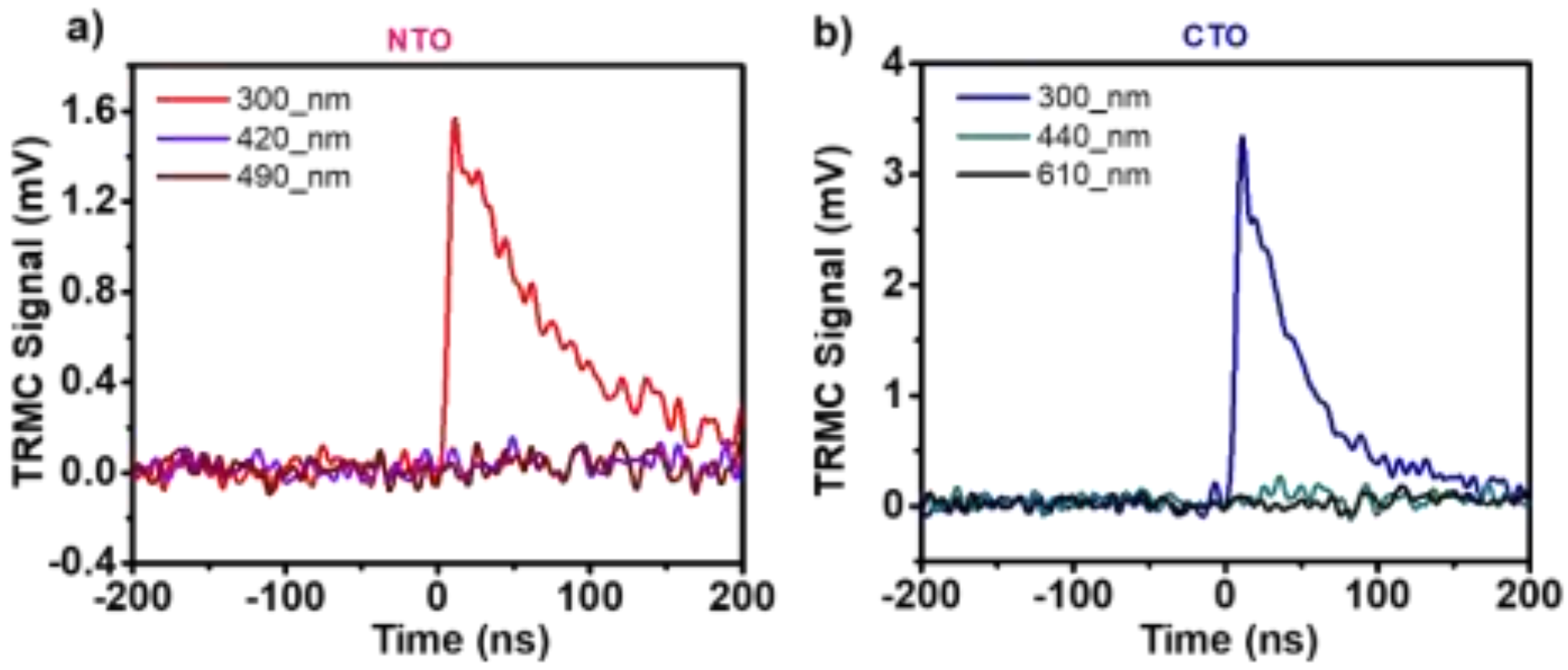

Figure S14: TRMC photoconductance transients of the (a) NTO and (b) CTO films under front illumination at excitation wavelengths in the ligand-field absorption region (NTO: 420 and 490 nm; CTO: 440 and 610 nm), compared with the corresponding charge-transfer response at 300 nm measured at the same photon fluence (6×10^{14} pulse^{-1} cm^{-2}). No transient photoconductivity above the noise level is observed under ligand-field excitation.

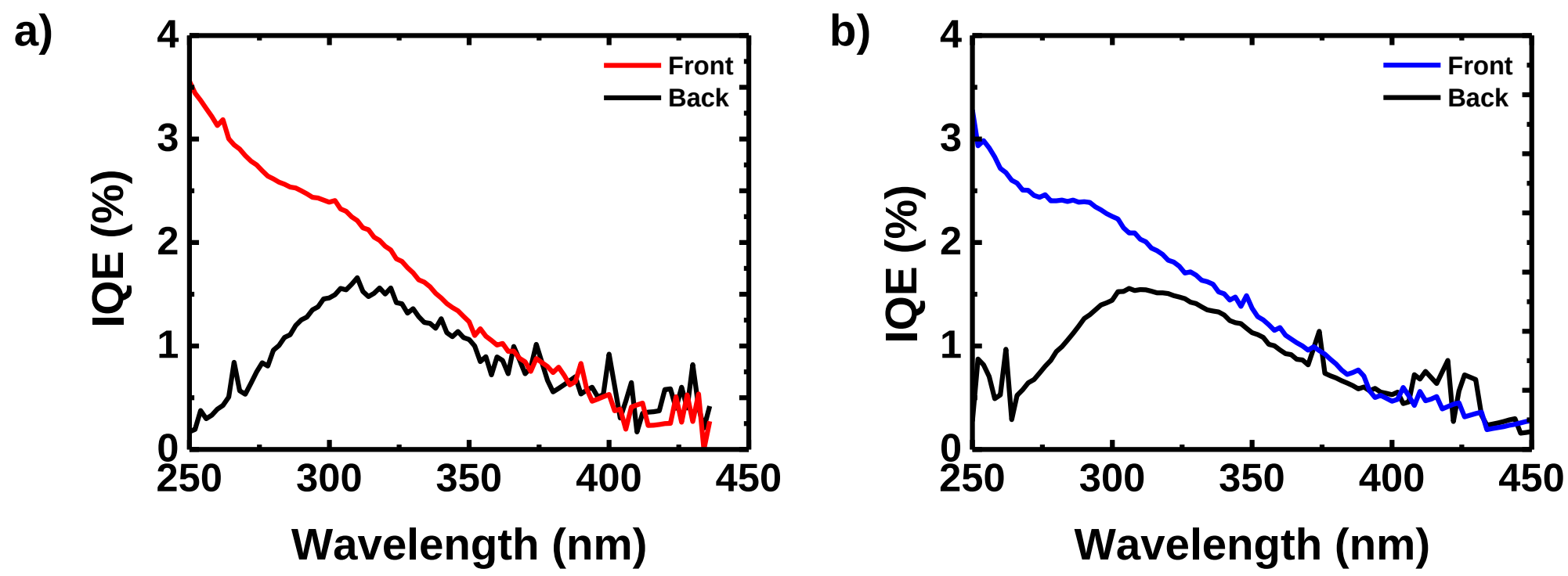

Figure S15: IQE of the (a) NTO and (b) CTO films measured under front (electrolyte-side) and back (substrate-side) illumination.
